# Outbreak investigations after identifying carbapenem-resistant *Pseudomonas aeruginosa*: a systematic review

**DOI:** 10.1186/s13756-023-01223-1

**Published:** 2023-04-03

**Authors:** Andrea C. Büchler, Selvi N. Shahab, Juliëtte A. Severin, Margreet C. Vos, Anne F. Voor in ’t holt

**Affiliations:** 1 Department of Medical Microbiology and Infectious Diseases, Erasmus MC University Medical Center, Rotterdam, The Netherlands; 2 Department of Clinical Microbiology, Dr. Cipto Mangunkusumo General Hospital - Faculty of Medicine, Universitas Indonesia, Jakarta, Indonesia

**Keywords:** *Pseudomonas aeruginosa*, Bacterial drug resistance, Outbreak investigations, Infection control, Systematic review

## Abstract

**Background:**

Carbapenem-resistant *Pseudomonas aeruginosa* (CRPA) are a serious cause of healthcare-associated infections. Part of the infection prevention and control measures are outbreak investigations (OI) of patients, healthcare workers (HCW), and the environment after identifying a CRPA in order to identify carriers and environmental reservoirs, so that targeted actions can be taken to prevent further transmission. However, little is known on when and how to perform such OI. Therefore, this systematic review aims to summarize OI performed after detection of CRPA in the endemic and epidemic hospital setting.

**Main text:**

Articles related to our research question were identified through a literature research in multiple databases (Embase, Medline Ovid, Cochrane, Scopus, Cinahl, Web of Science, and Google Scholar) until January 12, 2022 (Prospero registration number CRD42020194165). Hundred-twenty-six studies were included. In both the endemic and the epidemic setting, a median number of two out of seven predefined components of OI were identified. In the endemic setting, the most frequent component of OI was screening of the environment (28 studies, 62.2%). In the epidemic setting, screening of the environment (72 studies, 88.9%), and screening of patients during hospitalization (30 studies, 37%) were most frequently performed. Only 19 out of 126 studies (15.1%) reported screening of contact patients, and 37 studies reported screening of healthcare workers (HCW, 29.4%).

**Conclusion:**

Due to probable underreporting of OI in the literature, the available evidence for the usefulness of the individual components of OI is scarce. This could lead to inhomogeneous performance of OI after detection of CRPA in the healthcare setting, and with this, potential under- or overscreening. While we could show evidence for the usefulness for environmental screening in order to identify the mode of transmission, evidence for HCW screening is scarce and might not lead to the identification of modes of transmission. Further studies are needed to better understand CI in different settings and, finally, develop guidance on when and how to best perform OI.

**Supplementary Information:**

The online version contains supplementary material available at 10.1186/s13756-023-01223-1.

## Background

*Pseudomonas aeruginosa* is a well-known microorganism causing healthcare-associated infections (HAI), especially in immunocompromised patients or patients admitted to the intensive care unit (ICU) [1, 2]. The prevalence of multidrug-resistant (MDR) strains of *P. aeruginosa* was reported to be approximately 15% in the Unites States of America [3] and 17.2% in the European Union [4], but varies widely across and within countries and continents [5]. *P. aeruginosa* infections increased over the last two decades [5, 6], including bacteraemia and pneumonia, leading to high morbidity and mortality [7], and infections with MDR *P. aeruginosa* were no exception [8]. Carbapenem-resistant *P. aeruginosa* (CRPA) infections are of particular concern, since antimicrobial treatment of these bacteria is difficult [9], and mortality rates for bacteraemia are high (*i.e.* estimates between 30 and 70%) [7, 8, 10].

When a patient is unexpectedly identified with CRPA, infection prevention and control (IPC) measures should be taken. Especially in cases where contact precautions were not applied, an outbreak investigation (OI) as part of the IPC measures is a common approach to identify carriers and environmental reservoirs to stop further transmission. A OI may comprise several components, but usually contains the identification and screening of contact patients, as well as screening of the environment to identify environmental niches. Sometimes, screening of healthcare workers (HCW) is performed as well. OI are not well defined in the literature, except for tuberculosis [11], and more recently for COVID-19 [12]. In the review by Tomczyz et al. [13], the impact of practices to prevent and control CRPA were assessed, but not the impact of OI. Additionally, in 2014, the European Society of Clinical Microbiology and Infectious Diseases (ESCMID) released guidelines containing IPC measures meant to reduce transmission of MDR Gram-negative bacteria in hospitalized patients [14]. However, the guidelines do not give any recommendation regarding OI in either endemic or epidemic situations (Additional file 1: Table S1) but instead, specifically mention that there is no consensus on the role of screening to identify carriers. However, various screening strategies are used for carbapenem-resistant Gram-negatives, mostly in outbreak settings, as recently presented by Verdugo-Paiva et al. [15]. In addition, the guidelines recommend environmental cleaning and disinfection, nevertheless, no recommendation is made regarding screening of the environment to identify potential sources.

The routes of transmission of *P. aeruginosa* in the hospital setting often remain unknown [16], but several modes of transmission have been described in the literature, including hands of HCW [17], contaminated medical devices and supplies [18], and water-related devices [19]. These modes of transmission have been identified with rigorous OI using clinical, microbiological, and genotyping data. Nevertheless, there is little information available on if, when, and how a OI of patients, the environment, and HCW should be performed. Therefore, the aim of this systematic review is to give an overview of the strategies used for OI described in the literature, and their contribution to identify a source and/or contain the outbreak after detection of CRPA.

## Methods

This systematic review followed the guidelines presented in the Preferred Reporting Items for Systematic Reviews and Meta-Analyses (PRISMA) statement [20]. The PRISMA 2020 checklist is available in Additional file 1: document 1. Additionally, the protocol of the study is registered in the PROSPERO international prospective register of systematic reviews (CRD42020194165, Available from: https://www.crd.york.ac.uk/prospero/display_record.php?ID=CRD42020194165).

### Study selection

Articles related to our research question were identified through a literature research in Embase, Medline Ovid, Cochrane, Scopus, Cinahl, Web of Science, and Google Scholar (initial search until 10 June 2020, updated search performed on 12 January 2022). The search was not limited by language, date of publication, country of publication, or study design (Additional file 1: document 2).

Studies were included based on title and abstract if conducted in a hospital setting or long-term care facility (LTCF), and (1) reporting hospital or LTCF outbreaks involving CRPA, or (2) published case reports about CRPA. The specific inclusion criteria for the full-text selection was that the article should report on OI regarding CRPA*.* We excluded studies related to non-human infections, reviews, conference abstracts [21], and studies with missing information about OI after contacting the corresponding author. First, titles and abstracts of all retrieved citations were screened independently by ACB and SNS. After this screening, ACB and SNS performed a second screening based on the full-text. Disagreements were resolved by discussion with a third researcher (AFV) if necessary. We excluded duplicate publications. If several studies were published from the same author/institution during overlapping periods, we contacted the author/institution to avoid including duplicates. Reference lists of relevant reviews, which were identified during the literature research, were screened to identify additional studies that had been missed by our search strategy.

### Definitions and data extraction

Seven components of OI were predefined: (1) identification of contact patients (i.e. the exact definition of them), (2) screening of contact patients (i.e. the actually screened patients), (3) screening on admission, (4) screening during hospitalization, (5) screening of HCW, (6) screening of the environment (wet/dry), and (7) others.

A data extraction file was created, and the following data were extracted by ACB and SNS: first author, journal, year of publication, study site and location, type of hospital/healthcare setting, start and end date of the study, characteristics of the study, population (age, sex), number of patients with confirmed CRPA infection or colonization, number of patients selected for screening, criteria on which their selection was based, number of patients eventually screened and the results, number of HCW screened including screening sites and results, details of environment screening (e.g. number, location), number of performed components within a strategy, quality of the OI, and identification of the source and containment of the outbreak. In addition, we also extracted clinical outcomes, culture methods, and all molecular data if available. The data extraction file was sent to the corresponding authors of the included studies to verify the extracted data, and a request to provide missing data if applicable. In case we did not receive any response after the given deadline (*i.e.* 2 weeks), a reminder was sent. If no response was received and crucial information was missing, the study was excluded.

### Data analysis

Characteristics of the studies were collected in Excel. We conducted a qualitative synthesis of the OI components after detection of a CRPA. We stratified data by endemic/epidemic (as described by the authors of the included articles) setting, hospital type, and high/low prevalence of CRPA for studies conducted in Europe. Low and high prevalence was based on the EARS-Net data of CRPA from 2020, with low prevalence defined as < 10%, and high prevalence as ≥ 10% [22]. Continuous variables were analysed by median with range, or mean with standard deviation; categorical variables were analysed using percentages, or a median with range if applicable. P values were calculated using the chi-square or the independent sample median test. P values were only calculated if there was an expected count of more than five. All analyses were performed with IBM SPSS Statistics (Version 28.0.1.0).

### Study quality

Pre-specified quality assessment checklists according to the type of study were used to assess the risk of bias of the individual studies. ACB performed the quality assessments. The methodological quality was assessed for all included studies using the strengthening reporting of observational studies in epidemiology (STROBE) guideline [23], the guidelines for transparent reporting of outbreak reports and intervention studies of nosocomial infection (ORION) [24], or the consensus-based clinical case reporting (CARE) guideline [25] depending on the study design. The study quality was defined using the following threshold scores: STROBE (high 23–33 points, medium 12–22 points, and low 0–11 points), ORION (high 25–52 points, medium 18–34 points, and low 0–17 points), CARE (high 21–30 points, medium 11–20 points, and low 0–10 points). The classification was based on the achieved points of the maximum score possible, divided in thirds.

## Results

**Fig. 1:**
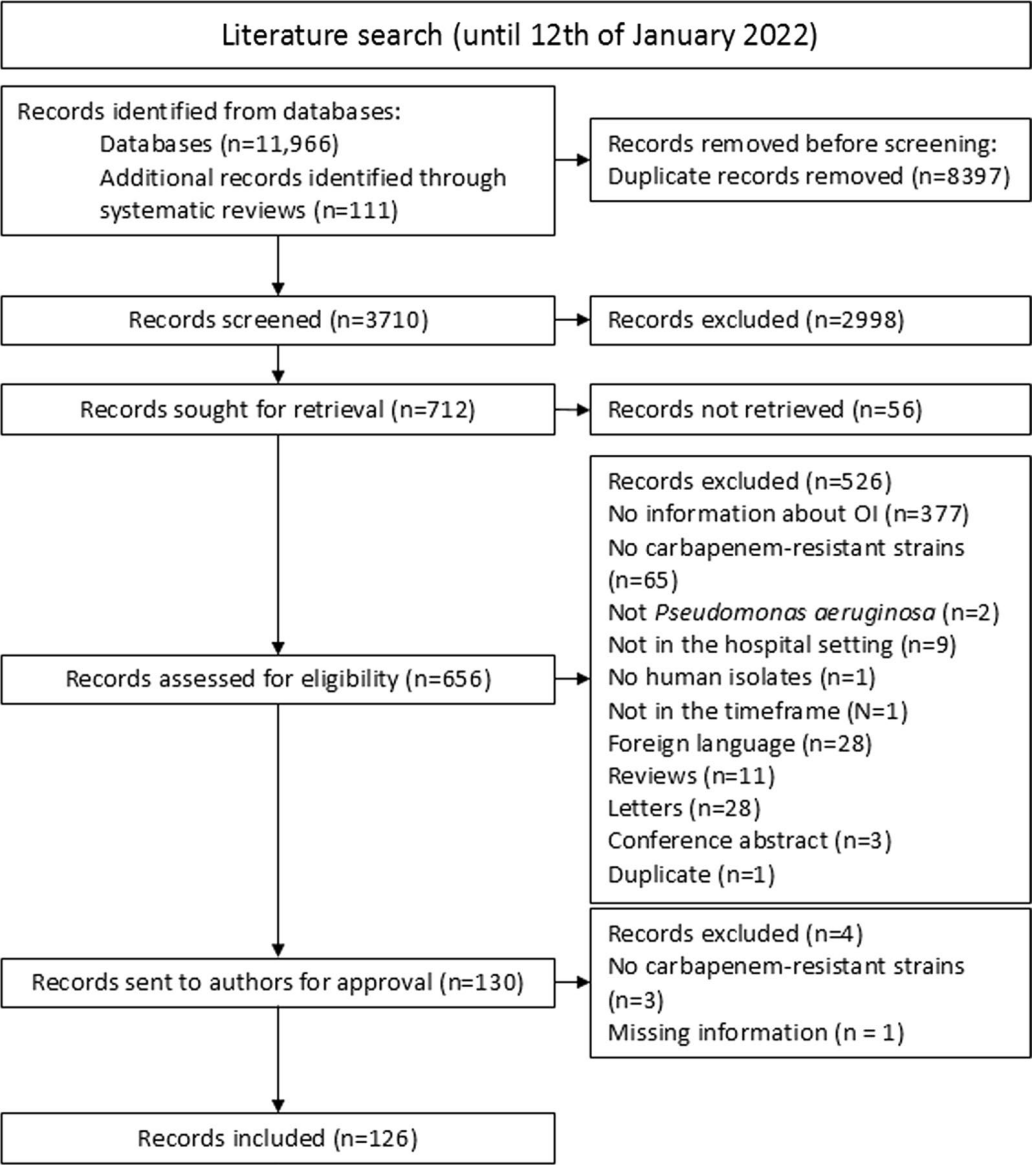
Flow chart of study selection according to the PRISMA guidelines. *OI* outbreak investigations

The literature search was performed on 12 January 2022 (Fig. 1). The search identified 3599 non-duplicate articles, and 111 additional articles were identified after searching the references list of reviews of interest. After the abstract selection, 712 articles were potentially relevant for this study. Full texts of these 712 articles were screened (56 full texts not retrieved), which resulted in 130 studies included of which data were extracted and forms sent to the authors (Fig. 1). The corresponding authors of 47 studies (36.2%) responded to our request to provide feedback on the extracted data. After review by the authors, four studies had to be excluded additionally, resulting in a total of 126 studies included (Fig. 1).

### Study characteristics

The included 126 studies were published between 1989 and 2021 (Additional file 1: Figure 1). Fifty-nine studies (46.8%) were conducted in Europe. The three most common countries of origin were France (n = 15, 11.9%), Germany (n = 11, 8.7%), and the Unites States (n = 10, 7.9%). Ninety-three of the studies were conducted at a tertiary care centre (73.8%), and two at a primary care centre, a secondary care centre, and in LTCF (1.6%). One study was from a rehabilitation centre. The precise setting was not described in 26 studies (20.6%), but all of them were performed in the hospital setting. Data on the number of beds was available for 75 studies (59.5%), resulting in a median of 925 beds per hospital (range 26–2974). Sixty-five outbreak descriptions (51.6%), 48 cohort studies (38.1%), six case–control studies (4.8%), three cross-sectional studies (2.4%), three case reports (2.4%), and one before-after study (0.8%) were included. Eighty-one studies were conducted in an epidemic setting (64.3%), while 47 were conducted in an endemic setting (37.3%) for CRPA as defined by the authors of the respective study. Four of the studies conducted in the endemic setting provided information about outbreaks during the study period and were therefore analysed in the epidemic group [26–29]. For one study, the setting was not described [30]. The median study size was 36 patients, ranging from 1 to 7071 patients. One study did not provide information on the study size [31]. Information about patients’ characteristics was fully available in 123 studies (97.6%): 114 studies provided information about adult patients (90.5%) and 24 studies about paediatric patients (19.0%, paediatric patients only: N = 9, 7.1%). ICU patients were included in 77 studies (61.1%), burn unit patients in 13 studies (10.3%, burn unit patients only: N = 5, 4.0%), cystic fibrosis (CF) patients in six studies (4.8%), paediatric ICU (PICU) patients in 10 studies (7.9%), and neonatal ICU (NICU) patients in seven studies (5.6%). The study duration was available for 117 (92.9%) of the studies providing a median of 17 months (SD ± 29.6 months). The characteristics of the included studies are available in Additional file 1: Table S2.

### Outbreak investigation components performed after identifying CRPA

In both the endemic and the epidemic setting, a median number of two out of seven predefined components of OI were identified (range 1–5, Table 1). The most common components in the endemic setting were screening of the environment in 62.2%, and screening of patients on admission and during the hospitalization in 46.7% and 46.7%, respectively. Identification and screening of contact patients was only described in less than 10% of the studies. In contrast, in the epidemic setting screening of the environment was more common (88.9%) as well as identifying (22.2%) and screening (19.8%) of contact patients than in the endemic setting. Nevertheless, patients’ screening on admission or during hospitalization was lower with 16.0% and 37.0%, respectively.

**Table 1:** Components of outbreak investigations after detection of CRPA stratified by endemic and epidemic setting as well as low and high prevalence for CRPA in European countries

	Endemic setting (N = 45)	Epidemic setting (N = 81)	P value	Low prevalence countries (N = 44)^1^	High prevalence countries (N = 8)^2^	P value
Median number of components (range)	2 (1–5)	2 (1–5)	0.697	2 (1–5)	1.5 (1–4)	0.833
*Components of outbreak investigations*						
1) Identifying contact patients (%)	4 (8.9)	18 (22.2)	0.059	9 (20.5)	2 (25.0)	–
2) Screening of contact patients (%)	3 (6.7)	16 (19.8)	**0.043**	8 (18.2)	2 (25.0)	–
3) Screening on admission (%)	21 (46.7)	13 (16.0)	** < 0.001**	15 (34.1)	1 (12.5)	–
4) Screening during hospitalization (%)	21 (46.7)	30 (37.0)	0.291	18 (40.9)	4 (50.0)	–
5) Screening of HCW (%)	12 (26.7)	25 (30.9)	0.620	13 (29.5)	0 (0)	–
6) Screening of the environment (%) Dry environment (%) Wet environment (%)	28 (62.2)^3^ 15 (33.3)^4^ 25 (55.6)^7^	72 (88.9) 38 (46.9)^5^ 68 (84.0)^6^	** < 0.001** 0.292 **0.002**	33 (75.0) 16 (36.4)^7^ 31 (70.5)^8^	7 (87.5) 3 (37.5) 7 (87.5)^6^	– – –
7) Other (%)	5 (11.1)	2 (2.5)	–	1 (2.3)	0 (0)	–

*CRPA* carbapenem-resistant *Pseudomonas aeruginosa*. *HCW* healthcare worker. Bold font indicates significant difference. –expected count lower than five, therefore, no P value was calculated

^1^Low prevalence was defined as < 10%, CRPA according to the EARS-Net [22]. Data on prevalence was only available for European countries

^2^high prevalence was defined as ≥ 10%, CRPA according to the EARS-Net [22], Data on prevalence was only available for European countries

^3^Information from one study missing

^4^information from five studies missing

^5^information from seven studies missing

^6^information from three studies missing

^7^information from four studies missing

^8^information from two studies missing

### Screening of contact patients

Nineteen out of 126 studies reported screening of contact patients. In these 19 studies, screening was performed for the following patients: patients who had contact with the patient identified with CRPA (n = 2), roommates of the CRPA positive patient (n = 3), patients admitted to the same ward at the same time as the CRPA positive patient (n = 9), patients hospitalized in the same clinic as the CRPA positive patient (n = 1), all hospitalized patients at a certain time point (n = 1), concomitantly admitted patients (n = 1), or patients undergoing the same procedure (n = 1). In one study, screening of relatives of the CRPA-positive patient was performed in addition [32]. The information was not available for two studies (10.5%). Only four out of 19 studies reported the outcome of the screening of contact patients: one found additional cases in 17.4% (15 out of 86 contact patients) [33], and the others found no additional cases [34–36] while screening 11 contact patients, 111 swabs of 52 contact patients, and 55 contact patients, respectively. Approaching contact patients after discharge was not described in any of the studies.

### Screening of HCW

Screening of HCW was performed in 37 (29.4%) of the studies, describing the screening of a median of 28.5 HCW per OI (range 5–120). In the majority of the studies (32; 86.5%), hands were cultured. No positive CRPA screenings were reported in 22 out of 37 studies (59.5%). Seven studies (18.9%) reported positive CRPA screenings of HCW with a range of one to 34 HCW per OI. In eight studies (21.6%), no results of the performed screening was reported. In 23 studies (62.2%) it was described that the isolates identified in HCW was non-identical to the isolate of the index patient, and eight studies (21.6%) reported identical isolates with a range of one to 27 identical isolates per OI. However, often it was not described how many HCW were screened and how many were identified with CRPA. In six studies (16.2%), no comparison of isolates was reported. Details of the HCW screening can be found in Additional file 1: Table S4.

### Screening of the environment

Overall, the environment was screened in 100 studies (79.4%); 53 (42.1%) screened the dry environment (e.g. surfaces, floors, equipment), and 93 (73.8%) screened the wet environment (e.g. water, sinks, showers, endoscopes, liquids). The quality of the reported results of the environmental screenings did not allow further analysis.

### Contribution of outbreak investigations to identify a source and contain the outbreak

In the epidemic setting, we analysed additional measures contributing to the containment of the outbreaks (Table 2). The most common measure was the construction of an epidemiological timeline in 64.2% of the studies, and a retrospective laboratory review to identify additional cases in 42.0%. In 61.7% of the studies, the source of the outbreak was identified, and in 81.5% the outbreak was contained. In all but three studies [35, 37, 38], contaminated environment was described as the primary source. The availability of an outbreak protocol was not described in any of the studies.

**Table 2:** Additional measures in the outbreak setting, source identification and containment of the outbreaks of the 81 studies conducted in an epidemic setting

Measures and outcome	Epidemic setting (N = 81)
*Measures*	
Outbreak protocol	0^1^
Epidemiological timeline (%)	52 (64.2)
Retrospective laboratory review (%)	34 (42.0)^1^
*Outcome outbreaks*	
Source identification/modes of transmission (%)	50 (61.7)^2^
Containment of the outbreak (%)	66 (81.5)^3^

*ERCP* endoscopic retrograde cholangiopancreatography

^1^information from one study missing

^2^information from five studies missing, Examples: contaminated medical devices and invasive procedures (duodenoscopes, bronchoscopes, transesophageal echocardiograph, retrograde urography), water-related (taps, sinks, showers, faucet aerators, toilet brush, hydrotherapy bath), contaminated products (body oil, breast milk feeding, basins, bite blocks), cross-transmission between patients

^3^information from seven studies missing

### Comparison of European studies by prevalence of the countries

Fifty-two (41.3%) out of the 126 reports were from Europe, and of these, 44 (84.6%) were from countries considered as low prevalence country, while 8 (15.4%) were classified as high prevalence. The median number of applied components of OI was similar in high prevalence countries compared to low prevalence countries (Table 1). However, the identification (25% vs. 20.5%) and screening (25% vs. 18.2%) of contact patients was performed more frequently, but not the screening on admission (12.5% vs. 34.1%). On the other hand, OI of the environment were performed more frequently in high prevalence countries (87.5% vs. 75%), especially of the wet environment (87.5% vs. 70.5%).

### Study quality assessment

In the endemic setting, the quality assessment of the 45 studies was performed with STROBE (n = 43) and ORION (n = 2). In the epidemic setting, STROBE was used for 12 studies, ORION for 66 studies, and CARE for 3 studies. Overall, study quality assessed with STROBE was high in 9.1%, moderate in 63.6%, and low in 27.3% of the studies. For ORION, high quality was 0%, moderate quality 44.1%, and low quality 55.9%. With CARE, all studies had moderate quality (100%) (Additional file 1: Table S5). In the endemic setting, the median STROBE score was 13 (range 6–25) and the median ORION score was 22 (range 22–22), in the epidemic setting the median STROBE score was 15.5 (range 10–24), the median ORION score was 17 [9–29], and the median CARE score was 16 (range 15–19), respectively.

## Discussion

### Summary of evidence

In this systematic review, analysing 126 studies, we found that a median of two OI components were performed in the endemic as well as in the epidemic setting. The most common OI components in the endemic settings were screening of the environment as well as general screening of patients on admission and during hospitalization. Identifying an environmental source might guide designated IPC measures to prevent further spread. Measures to combat spread of CRPA were previously described by a systematic review by Tomczyk et al. [13], where the most frequent IPC measures were contact precautions, active surveillance cultures (from all patients, high risk patients, and also contact patients of indexes), monitoring, audit and feedback of preventive measures, and patient cohorting. However, this review did not describe all possible components of OI, even though environmental cleaning was mentioned in 40% of the studies, which implies previous environmental screening might have been performed. Environmental screening is a labour-intensive measure, but to the best of our knowledge, no cost-effectiveness analysis is available in the literature. It is a balance in which it takes an effort to identify an environmental source, but the identification will possibly prevent a potential (ongoing) outbreak. The same applies for screening of patients at admission or during hospitalization. Depending on the prevalence of CRPA in the specific healthcare setting, the costs might exceed the benefits and index-case driven screening might be more appropriate. Recent studies show a benefit of overall screening in the detection of carbapenem-resistant Enterobacterales [39, 40], but it remains unknown if this is also applicable for *P. aeruginosa*.

In the epidemic setting, screening of the environment as well as identification and screening of contact patients were the most commonly performed components. However, identification and screening of contact patients was only reported in one fifth of the studies. As the epidemic studies report successful containment of the outbreaks in four out of five outbreaks, it remains unknown whether these components were not reported or actually not performed. Our results are in addition to a recently published systematic review by Verdugo-Paiva et al. [15], which reported mostly screening of high-risk patients for carbapenem-resistant Gram-negatives in the endemic as well as the epidemic setting, whereas screening of contact patients was only described in less than half of the studies. In addition, the definition of contact patients was often vague in these studies, ranging from roommates to ward mates and to hospitalized at the same time in the whole hospital or admitted at the same time. These inhomogeneous definitions complicate the interpretation of the yield of screening of contact patients, and therefore, also the clinical impact. Interestingly, the identification and screening of contact patients were more often performed in high prevalence European countries compared to low prevalence countries, which might be due to the expected higher yield in countries with a higher prevalence overall or publication bias due to underreporting of OI without any secondary cases. For other carbapenem-resistant Gram-negative microorganisms, such as Enterobacterales, a few studies report the identification and screening of contact patients with a yield of 2.0% in a low endemic setting [41], but this result might not be transferable to *P. aeruginosa* due to the often water-related environmental niches [42]*.*

Screening of HCW is often discussed as an additional measure to identify the mode of transmission, but guidance on if and when this would be useful, and how this should be performed, is scarce. Less than a third of the studies reported screening of HCW, often without mentioning the screening site(s) or the result of the screening. If screening sites were reported, it was mainly performed on the hands of HCW. However, detection of CRPA on the hands of HCW is difficult to interpret, as it could be a transient colonization while working with patients colonized with CRPA or an actual, more persistent, source of transmission from a colonized HCW to a patient or the environment. Only two studies reported screening of HCW by rectal swabs or stool cultures [43, 44], interestingly without any detection of CRPA (Additional file 1: Table S3). Nevertheless, screening of HCW is often performed as part of a bundle to increase awareness for hand hygiene.

### Implications for clinical practice and research

Due to the probable underreporting of OI in the literature, the available evidence for the individual components is scarce. This leads to an inhomogeneous performance of OI after detection of CRPA in the healthcare setting, and with this, potential under- or overscreening. The current ESCMID guidelines on management of the infection control measures to reduce transmission of multidrug-resistant Gram-negative bacteria in hospitalized patients recommends the enforcement of hand hygiene, and contact precautions in the case of multidrug-resistant *P. aeruginosa* in the endemic setting [14]. In the epidemic setting, emphasizing hand hygiene, introducing contact precautions for colonized patients, and implementing active screening at hospital admission are recommended (Additional file 1: Table S1) [14]. Guidance for how to perform OI, especially identification and screening of contact patients, but also measures for source identification, is urgently needed to standardize and optimize the yield of OI. In addition, the definition of contact patients is inhomogeneous, making it challenging to compare the different studies. For this, prospective studies focusing on how to conduct OI are needed and an implementation of OI in the ORION statement would be beneficious [24].

### Strengths and limitations

Our systematic review gives a detailed overview over OI performed after detection of CRPA with focus on individual components of OI. This is an underreported and underappreciated topic in the current literature, even though it influences daily practice of IPC teams worldwide. It has some limitations. First, in the included studies, the patients’ characteristics are poorly described. Often not even basic demographic information such as age and gender are available. This might make it more difficult to apply the generated knowledge to specific patient populations. Second, the response rate of the authors to review the extracted data was only one-third, partly caused by the gaps between publication date and performance of the search, but probably also due to the pandemic with SARS-CoV-2. This might have caused a lack of information and data in our review. Third, as it is an overall view, there is a mix of high and low prevalence settings, which might have influenced the OI performed. Therefore, we analysed the data by prevalence of CRPA in the respective country, but this data was only available for European countries. Fourth, we looked at OI from the IPC perspective for the healthcare facility only, and not from the perspective of the individual patient.

## Conclusions

In conclusion, OI are poorly described in the literature and further studies are needed to better define and evaluate OI after identifying CRPA in the endemic as well as the epidemic setting. While we could show evidence for the usefulness for environmental screening in order to identify the mode of transmission, evidence for HCW screening is scarce and might not lead to the identification of modes of transmission. Even though the yield of screening of CP is poorly described in the literature, it might still be key to contain an outbreak. International guidelines should be further improved and include recommendations for OI as well as guidance to report the OI performed in future studies.

## Supplementary Information

**Additional file 1**.** Table 1**. Overview table of the evidence and recommendation for multidrug-resistant Pseudomonas aeruginosa in the healthcare setting, adapted from the ESCMID guidelines for the management of the infection control measures to reduce transmission of multidrug-resistant Gram-negative bacteria in hospitalized patients.** Table 2**. Study characteristics of the included 126 studies.** Table 3**. Outbreak investigations after detection of CRPA stratified by hospital setting. **Table 4**. Quality assessments of the included studies by study type.** Document 1**. PRISMA 2020 Checklist.** Document 2**. Literature search strategies.** Figure 1**. Year of publication of the included studies (N=126).

## Data Availability

All data generated or analysed during this study are included in the article and its additional files. The data extraction file is available on request.

## Abbreviations

CARE guidelines: Case reports guidelines
CF: Cystic fibrosis
CRPA: Carbapenem-resistant *Pseudomonas aeruginosa*
ESCMID: European Society for Clinical Microbiology and Infectious Diseases
HAI: Healthcare-associated infections
HCW: Healthcare workers
ICU: Intensive care unit
IPC: Infection prevention and control
LTCF: Long term care facility
MDR: Multidrug-resistant
NICU: Neonatal intensive care unit
ORION statement: Guidelines for transparent reporting of outbreak reports and intervention studies of nosocomial infection
OI: Outbreak investigations
PICU: Paediatric intensive care unit
PRISMA statement: Preferred reporting items for systematic reviews and meta-analysis statement
SARS-CoV-2: Severe acute respiratory syndrome coronavirus type 2
STROBE: The strengthening the reporting of observational studies in epidemiology
