# Supplementary material for: Outbreak investigations after identifying carbapenem-resistant Pseudomonas aeruginosa: a systematic review
Source: Antimicrob Resist Infect Control. 2023 Apr 3;12:28. doi: 10.1186/s13756-023-01223-1 (PMC10068724; doi:10.1186/s13756-023-01223-1)

**Additional material**

**Additional Table 1.** Overview table of the evidence and recommendation for multidrug-resistant *Pseudomonas aeruginosa* in the healthcare setting, adapted from the ESCMID guidelines for the management of the infection control measures to reduce transmission of multidrug-resistant Gram-negative bacteria in hospitalized patients.^1^

| Measure | Evidence | Recommendation |
| --- | --- | --- |
| Endemic situations | | |
| Hand hygiene | Moderate | Strong |
| Contact precautions (CP) | Moderate | Strong |
| Environmental Cleaning | Moderate | Conditional |
| Antimicrobial stewardship program | Moderate | Conditional |
| Educational programs for healthcare workers | Moderate | Conditional |
| Alert code (previous positive) and pre-emptive CP | Not available | Not available |
| Isolation room | Not available | Conditional |
| Infection prevention and control infrastructure | Not available | Not available |
| Epidemic situations | | |
| Emphasizing hand hygiene | Very low | Strong |
| Introducing CP for colonized patients | Very low | Strong |
| Implementing active screening cultures at hospital admission | Very low | Strong |
| Implementing regular environmental cleaning and monitoring of it | Moderate | Conditional |
| Environmental screening | Low | Conditional |
| Antimicrobial stewardship program | Very low | Conditional |
| Educational programs for healthcare workers | Very low | conditional |
| Alert code (previous positive) and pre-emptive CP | Very low | conditional |
| Cohort patients | Very low | Strong |
| Cohort staff | Very low | Strong |
| Isolation room | Low | Strong |
| Healthcare workers screening | Not available | Not available |
| Chlorhexidine gluconate for patients bathing | Not available | Not available |
| Infection prevention and control infrastructure | Not available | Not available |

^1^Tacconelli E. CMA, Dancer S.J., De Angelis G., Falcone M., Frank U., Kahlmeter G., Pan A., Petrosillo N., Rodriguez-Bano J., Singh N., Venditti M., Yokoe D.S., Cookson B. ESCMID guidelines for the management of the infection control measures to reduce transmission of multidrug-resistant Gram-negative bacteria in hospitalized patients. Clin Microbiol Infect **2014**;20 (Suppl. I):1-55.

**Additional Table 2.** Study characteristics of the included 126 studies.

| Study | Year of publication | Country | Study period begin | Study period end | Study design | outbreak | Number of OI components | Identifying contact patients | screening of contact patients | screening on admission | screening during hospitalization | screening of HCW | Screening environment | other | STROBE | ORIOIN | CARE |
| --- | --- | --- | --- | --- | --- | --- | --- | --- | --- | --- | --- | --- | --- | --- | --- | --- | --- |
| Achour (1) | 2006 | Tunisia | 01.10.2004 | 31.01.2005 | outbreak | yes | 2 | no | no | no | no | yes | yes | no |  | 12 |  |
| Adachi (2) | 2009 | USA | 01.05.2001 | 30.04.2005 | prospective cohort study | yes | 5 | yes | yes | yes | yes | no | yes | no | 11 |  |  |
| Aditi (3) | 2017 | India | 01.01.2015 | 31.03.2016 | prospective cohort study | no | 4 | no | no | yes | yes | yes | yes | no | 15 |  |  |
| Agodi (4) | 2007 | Italy | 01.01.2004 | 31.07.2004 | prospective cohort study | no | 1 | no | no | yes | no | no | no | no | 20 |  |  |
| Aguilera-Saez (5) | 2019 | Spain | 01.04.2016 | 30.09.2019 | outbreak | yes | 2 | no | no | no | yes | no | yes | no |  | 22 |  |
| Alipour (6) | 2017 | Turkey | 01.10.2013 | 31.12.2013 | outbreak | yes | 2 | no | no | no | no | yes | yes | no |  | 15 |  |
| Ambrogi (7) | 2016 | France | 01.08.2014 | 28.02.2015 | outbreak | yes | 1 | no | no | no | no | no | yes | no |  | 11 |  |
| Araoka (8) | 2014 | Japan | 01.05.2008 | 31.05.2013 | prospective cohort study | no | 2 | yes | yes | no | no | no | no | no | 7 |  |  |
| Aubron (9) | 2005 | France | 01.09.1995 | 31.03.1999 | prospective cohort study | no | 3 | no | no | yes | yes | no | yes | no | 6 |  |  |
| Azimi (10) | 2019 | Iran | 2013 | 2013 | prospective cohort study | no | 1 | no | no | no | no | no | yes | no | 6 |  |  |
| Bertrand (11) | 2000 | France | 01.05.1997 | 31.01.2000 | outbreak | yes | 4 | no | no | yes | yes | yes | yes | no |  | 17 |  |
| Bertrand (12) | 2001 | France | 01.01.1998 | 31.12.1999 | prospective cohort study | no | 2 | no | no | yes | yes | no | no | no | 8 |  |  |
| Bilavsky (13) | 2013 | Israel | 01.04.2009 | 31.10.2011 | outbreak | yes | 1 | no | no | no | no | no | yes | no |  | 9 |  |
| Bingen (14) | 1996 | France/  USA | 01.12.1993 | 31.10.1994 | outbreak | yes | 1 | no | no | no | no | no | yes | no |  | 17 |  |
| Biswal (15) | 2014 | India | na | na | prospective cohort study | no | 4 | no | no | yes | yes | yes | yes | no | 13 |  |  |
| Boutiba-Ben Boubaker (16) | 2003 | Tunisia | 01.09.1999 | 30.11.1999 | outbreak | yes | 1 | no | no | no | no | no | yes | no |  | 16 |  |
| Bradbury (17) | 2009 | Australia | na | na | prospective cohort study | no | 1 | no | no | no | no | no | yes | no | 9 |  |  |
| Breathnach (18) | 2012 | UK | 2005 | 2011 | outbreak | yes | 2 | yes | na | no | no | no | yes | no |  | 15 |  |
| Buhl (19) | 2019 | Germany | na | na | outbreak | yes | 4 | yes | yes | no | yes | no | yes | no |  | 15 |  |
| Bukholm (20) | 2002 | Norway | 01.12.1999 | 30.09.2000 | outbreak | yes | 2 | no | no | no | yes | no | yes | no |  | 23 |  |
| Carattoli (21) | 2013 | Italy | 01.05.2013 | 31.05.2013 | case report | yes | 4 | yes | yes | no | no | yes | yes | no |  |  | 19 |
| Catho (22) | 2021 | Switzerland | 01.01.2018 | 30.09.2019 | outbreak | yes | 3 | no | no | yes | yes | no | yes | no |  | 26 |  |
| Cezario (23) | 2009 | Brazil | 01.11.2003 | 30.06.2005 | retrospective case-control study | yes | 4 | yes | yes | no | no | yes | yes | no | 17 |  |  |
| Chaves (24) | 2017 | Brazil | 01.12.2011 | 31.01.2013 | retrospective case-control study | yes | 3 | no | no | no | yes | yes | yes | no | 20 |  |  |
| Cholley (25) | 2010 | France | 01.10.2007 | 30.09.2008 | prospective cohort study | yes | 1 | no | no | no | yes | no | no | no | 10 |  |  |
| Ciofi degli Atti (26) | 2014 | Italy | 01.03.2011 | 31.12.2012 | outbreak | yes | 5 | yes | yes | yes | yes | no | yes | no |  | 17 |  |
| Corona )-Nakamura (27 | 2001 | Mexico | 01.04.1998 | 31.05.1998 | prospective cohort study | no | 2 | no | no | no | no | yes | yes | no | 11 |  |  |
| Cortes (28) | 2009 | Colombia | 01.05.2001 | 31.12.2004 | outbreak | yes | 3 | no | no | yes | yes | no | yes | no |  | 20 |  |
| Corvec (29) | 2008 | France | 01.01.1996 | 31.12.2004 | outbreak | yes | 1 | no | no | no | yes | no | no | no |  | 13 |  |
| Crespo (30) | 2004 | Colombia/  UK | 01.02.1996 | 30.11.2003 | outbreak | yes | 2 | no | no | no | no | yes | yes | no |  | 16 |  |
| Crivaro (31) | 2009 | Italy | 01.07.2005 | 30.06.2007 | prospective cohort study | yes | 3 | no | no | no | yes | yes | yes | no | 13 |  |  |
| DalBen (32) | 2013 | Brazil | 01.04.2000 | 31.07.2002 | prospective cohort study | no | 2 | no | no | yes | yes | no | no | no | 14 |  |  |
| De Jonge (33) | 2019 | Netherlands | 01.12.2010 | 31.07.2018 | prospective cohort study | no | 1 | no | no | no | no | no | yes | no | 13 |  |  |
| Decraene (34) | 2018 | UK | 01.11.2015 | 30.09.2017 | outbreak | yes | 4 | yes | na | yes | yes | no | yes | no |  | 17 |  |
| Deplano (35) | 2005 | Belgium | 01.07.2002 | 31.12.2002 | outbreak | yes | 3 | no | no | yes | no | yes | yes | no |  | 17 |  |
| Döring (36) | 1996 | Germany | 01.07.1992 | 31.08.1992 | prospective cohort study | no | 2 | no | no | no | no | yes | yes | no | 13 |  |  |
| Dubois (37) | 2001 | France | 01.06.1995 | 31.03.1998 | outbreak | yes | 2 | no | no | no | no | yes | yes | no |  | 15 |  |
| Dunne (38) | 2021 | USA | 01.03.2019 | 30.09.2019 | outbreak | yes | 1 | no | no | no | no | no | yes | no |  | 21 |  |
| Dwivedi (39) | 2009 | India | 01.07.2005 | 30.06.2007 | prospective cohort study | no | 3 | no | no | no | yes | yes | yes | no | 13 |  |  |
| Elias (40) | 2009 | Germany | 01.11.2007 | 31.12.2007 | outbreak | yes | 1 | no | no | no | no | no | yes | no |  | 21 |  |
| Fernandez-Cuenca (41) | 2020 | Spain | 01.04.2016 | 31.08.2017 | outbreak | yes | 1 | no | no | no | no | no | yes | no |  | 17 |  |
| Ferreira (42) | 2004 | Brazil | 01.12.1999 | 31.03.2001 | prospective cohort study | no | 2 | no | no | yes | yes | no | no | no | 14 |  |  |
| Flateau (43) | 2012 | France | 01.03.2012 | 31.03.2012 | case report | yes | 2 | yes | yes | no | no | no | no | no |  |  | 15 |
| Fraser (44) | 2004 | USA | 01.07.2002 | 31.08.2002 | outbreak | yes | 2 | no | no | no | no | no | yes | yes |  | 15 |  |
| Freire (45) | 2021 | Brazil | 01.02.2019 | 28.02.2020 | outbreak | yes | 2 | no | no | yes | yes | no | no | no |  | 24 |  |
| Furtado (46) | 2006 | Brazil | 1997 | 2003 | prospective cohort study | no | 2 | yes | yes | no | no | no | no | no | 13 |  |  |
| Galdys (47) | 2019 | USA | 01.07.2014 | 31.12.2014 | outbreak | yes | 3 | yes | yes | no | no | no | yes | no |  | 18 |  |
| Gbaguidi-Haore (48) | 2018 | France | 2009 | 2013 | prospective outbreak description | yes | 3 | no | no | yes | yes | no | yes | no |  | 22 |  |
| Geladari (49) | 2017 | Greece | 01.03.2012 | 31.07.2014 | prospective cohort study | no | 1 | no | no | no | yes | no | no | no |  | 22 |  |
| Gibb (50) | 2002 | Canada | 1995 | 1997 | outbreak | yes | 1 | no | no | no | no | no | yes | no |  | 13 |  |
| Gomes (51) | 2011 | Brazil | 01.04.2002 | 28.02.2007 | prospective cohort study | yes | 4 | yes | yes | no | no | yes | yes | no |  | 20 |  |
| Goudarzi Ghazi (52) | 2012 | Iran | na | na | prospective cohort study | no | 1 | no | no | no | no | no | yes | no | 10 |  |  |
| Griffith (53) | 1989 | USA | 01.07.1986 | 31.12.1986 | prospective cohort study | no | 4 | no | no | yes | yes | yes | yes | no | 14 |  |  |
| Gülay (54) | 2001 | Turkey | 01.01.1997 | 31.07.1997 | retrospective case-control study | yes | 2 | no | no | no | no | yes | yes | no | 15 |  |  |
| Harris (55) | 2011 | USA | 01.09.2001 | 30.09.2006 | prospective cohort study | no | 3 | no | no | yes | yes | no | no | yes | 16 |  |  |
| Hopman (56) | 2019 | Netherlands | 01.02.2018 | 28.02.2018 | outbreak | yes | 3 | yes | yes | no | no | no | yes | no |  | 14 |  |
| Hota (57) | 2009 | Canada | 01.12.2004 | 28.02.2007 | outbreak | yes | 4 | yes | yes | no | yes | no | yes | no |  | 20 |  |
| Hsueh (58) | 1998 | Taiwan | 01.04.1997 | 31.05.1997 | outbreak | yes | 4 | yes | yes | no | no | yes | yes | no |  | 18 |  |
| Hu (59) | 2017 | China | 01.03.2014 | 31.08.2014 | prospective cohort study | no | 2 | no | no | yes | no | no | yes | no | 17 |  |  |
| Inglis (60) | 2010 | Australia | 2006 | 2008 | outbreak | yes | 2 | no | no | no | no | yes | yes | no |  | 17 |  |
| Kanayama (61) | 2016 | Japan | 01.01.2013 | 31.01.2015 | outbreak | yes | 4 | yes | yes | no | yes | no | yes | no |  | 26 |  |
| Karami (62) | 2019 | Iran | 01.07.2016 | 30.04.2017 | cross-sectional | no | 1 | no | no | no | no | no | yes | no | 13 |  |  |
| Kateete (63) | 2016 | Uganda | 01.02.2007 | 30.09.2009 | cross-sectional | no | 1 | no | no | no | no | no | yes | no | 13 |  |  |
| Kikuchi (64) | 2007 | Japan | 01.07.2004 | 31.08.2004 | outbreak | yes | 1 | no | no | no | no | no | yes | no |  | 13 |  |
| Knoester (65) | 2014 | Netherlands | 01.02.2009 | 31.01.2012 | outbreak | yes | 4 | yes | yes | no | yes | no | yes | no |  | 17 |  |
| Kohlenberg (66) | 2010 | Germany | 01.01.2006 | 31.10.2006 | outbreak | yes | 1 | no | no | no | no | no | yes | no |  | 29 |  |
| Kossow (67) | 2017 | Germany | 2012 | 2016 | prospective cohort study | no | 3 | no | no | yes | yes | yes | no | no | 13 |  |  |
| Kouda (68) | 2011 | Japan | 2005 | 2007 | outbreak | yes | 1 | no | no | no | no | no | yes | no | 11 |  |  |
| Kousouli (69) | 2018 | Greece | 01.01.2010 | 31.12.2015 | prospective interventional study | no | 4 | yes | yes | yes | yes | no | no | no | 19 |  |  |
| Kovaleva (70) | 2009 | Netherlands | 01.07.2008 | 31.10.2008 | outbreak | yes | 1 | no | no | no | no | no | yes | no |  | 12 |  |
| Kristie Johnson (71) | 2009 | USA | 2001 | 2006 | prospective cohort study | no | 3 | no | no | yes | yes | no | no | yes | 20 |  |  |
| Kumarage (72) | 2019 | UK | 01.07.2017 | 31.12.2017 | outbreak | yes | 1 | no | no | no | no | no | yes | no |  | 17 |  |
| Ling (73) | 2013 | Singapore | 01.01.2009 | 31.03.2009 | outbreak | yes | 1 | no | no | no | no | no | yes | no |  | 17 |  |
| Liu (74) | 2018 | China | 2012 | 2017 | prospective cohort study | no | 3 | no | no | no | yes | yes | yes | no | 15 |  |  |
| Loconsole (75) | 2020 | Italy | 01.08.2019 | 30.11.2019 | case report | yes | 3 | no | no | no | no | yes | yes | yes |  |  | 16 |
| Lolans (76) | 2005 | USA | 01.05.2003 | 31.10.2004 | outbreak | yes | 1 | no | no | no | yes | no | no | no |  | 11 |  |
| Lyytikäinen (77) | 2001 | Finland | 01.08.1993 | 31.10.1995 | outbreak | yes | 1 | no | no | no | no | no | yes | no |  | 16 |  |
| Machida (78) | 2014 | Japan | 01.04.2006 | 31.08.2009 | outbreak | yes | 1 | no | no | no | no | no | yes | no |  | 26 |  |
| Mahmoud (79) | 2020 | Egypt | 01.12.2017 | 31.01.2020 | prospective cohort study | na | 2 | no | no | no | no | yes | yes | no | 10 |  |  |
| Mammina (80) | 2008 | Italy | 01.01.2003 | 31.01.2004 | prospective cohort study | yes | 1 | no | no | no | yes | no | no | no | 26 |  |  |
| Matar (81) | 2005 | Lebanon | 01.09.2003 | 31.05.2004 | prospective cohort study | no | 1 | no | no | no | no | no | yes | no | 7 |  |  |
| Mayr (82) | 2017 | Austria | 01.08.2012 | 30.09.2012 | outbreak | yes | 3 | no | no | yes | no | yes | yes | no |  | 17 |  |
| Mendes (83) | 2021 | Brazil | 01.01.2012 | 31.12.2012 | prospective cohort study | no | 2 | no | no | yes | yes | no | no | no | 19 |  |  |
| Mentzelopoulos (84) | 2007 | Greece | 01.09.2005 | 31.10.2008 | prospective case-control study | yes | 3 | no | no | no | yes | yes | yes | no | 24 |  |  |
| Milan (85) | 2013 | France | October | January | outbreak | yes | 3 | yes | yes | no | no | no | yes | no |  | 16 |  |
| Miranda (86) | 2001 | Mexico | 01.09.1997 | 31.10.1997 | outbreak | yes | 3 | no | no | no | yes | yes | yes | no |  | 22 |  |
| Moremi (87) | 2017 | Tanzania | 01.12.2014 | 30.09.2015 | prospective cohort study | no | 3 | no | no | yes | no | no | yes | yes | 18 |  |  |
| Mudau (88) | 2013 | South Africa | 01.01.2009 | 31.01.2011 | outbreak | yes | 3 | no | no | no | yes | yes | yes | no |  | 25 |  |
| Muthu (89) | 2006 | India | 01.05.2003 | 31.05.2004 | prospective cohort study | no | 1 | no | no | no | no | no | yes | no | 13 |  |  |
| Nagao (90) | 2011 | Japan | 2004 | 2010 | outbreak | yes | 2 | no | no | no | yes | no | yes | no |  | 22 |  |
| Orsi (91) | 1994 | Italy | 01.06.1991 | 31.08.1992 | prospective cohort study | no | 1 | no | no | no | no | no | yes | no | 13 |  |  |
| Ozer (92) | 2009 | Turkey | na | na | prospective cohort study | no | 3 | no | no | yes | yes | yes | no | no | 11 |  |  |
| Panzig (93) | 1999 | Germany | 01.12.1996 | 31.05.1998 | outbreak | yes | 2 | no | no | no | no | yes | yes | no |  | 10 |  |
| Parcell (94) | 2018 | UK | 01.01.2012 | 31.05.2013 | outbreak | yes | 1 | no | no | no | no | no | yes | no |  | 18 |  |
| Pelegrin (95) | 2019 | Indonesia | na | na | prospective before-after-study | no | 2 | no | no | no | no | yes | yes | no |  | 22 |  |
| Pena (96) | 2003 | Spain | 01.02.1998 | 30.09.2000 | outbreak | yes | 3 | yes | yes | no | no | no | yes | no |  | 20 |  |
| Pena (97) | 2007 | Spain | 01.05.2003 | 30.06.2004 | prospective cohort study | no | 2 | no | no | yes | yes | no | no | no | 23 |  |  |
| Pirnay (98) | 2003 | Belgium | 01.07.1998 | 31.07.1999 | retrospective cohort study | yes | 3 | no | no | yes | no | yes | yes | no | 16 |  |  |
| Pitten (99) | 2001 | Germany | 01.01.1997 | 31.03.1998 | outbreak | yes | 2 | no | no | no | no | yes | yes | no |  | 17 |  |
| Prashant (100) | 2010 | India | 01.01.2003 | 31.08.2004 | prospective cohort study | no | 1 | no | no | no | no | no | yes | no | 14 |  |  |
| Quick (101) | 2014 | UK | na | na | prospective cohort study | no | 2 | no | no | yes | no | no | yes | no | 16 |  |  |
| Ramirez (102) | 2013 | Argentina | 01.01.2005 | 30.04.2012 | cohort study | no | 1 | no | no | no | yes | no | no | no | 10 |  |  |
| Richard (103) | 1994 | France | 01.01.1990 | 31.12.1992 | outbreak | yes | 1 | no | no | no | no | no | yes | no |  | 27 |  |
| Ruiz (104) | 2004 | Spain | na | na | retrospective cohort study | no | 1 | no | no | no | no | no | yes | no | 8 |  |  |
| Sader (105) | 1993 | Brazil | 01.06.1991 | 31.08.1991 | outbreak | yes | 2 | no | no | no | no | yes | yes | no |  | 15 |  |
| Saharman (106) | 2019 | Indonesia | 01.04.2013 | 31.08.2014 | prospective cohort study | no | 5 | no | no | yes | yes | yes | yes | yes | 24 |  |  |
| Salauze (107) | 1997 | France | 01.11.1995 | 31.01.1996 | outbreak | yes | 3 | yes | yes | no | no | no | yes | no |  | 16 |  |
| Salimi (108) | 2010 | Iran | 01.02.2008 | 30.06.2008 | prospective cohort study | no | 1 | no | no | no | no | no | yes | no | 13 |  |  |
| Salm (109) | 2016 | Germany | 01.01.2012 | 30.04.2014 | retrospective case-control study | yes | 1 | no | no | no | no | no | yes | no |  | 26 |  |
| Schäfer (110) | 2019 | Germany | 2015 | 2017 | retrospective cohort study | no | 2 | yes | no | yes | no | no | no | no | 17 |  |  |
| Seki (111) | 2013 | Japan | 2004 | 2012 | outbreak | yes | 2 | no | no | no | yes | no | yes | no |  | 21 |  |
| Sekiguchi (112) | 2007 | Japan | 01.09.2004 | 31.05.2005 | outbreak | yes | 1 | no | no | no | no | no | yes | no |  | 16 |  |
| Shigemura (113) | 2015 | Japan | 01.07.2007 | 31.08.2008 | outbreak | yes | 1 | no | no | no | yes | no | no | no |  | 18 |  |
| Snyder (114) | 2013 | UK | 01.01.2002 | 31.12.2007 | outbreak | yes | 1 | no | no | no | no | no | yes | no |  | 14 |  |
| Sorin (115) | 2001 | USA | 01.08.1998 | 31.10.1998 | outbreak | yes | 1 | no | no | no | no | no | yes | no |  | 17 |  |
| Suarez (116) | 2011 | Spain | 01.01.2006 | 31.05.2008 | prospective cohort study | yes | 2 | no | no | no | yes | no | yes | no | 13 |  |  |
| Talon (117) | 1995 | France | 01.09.1991 | 31.10.1991 | outbreak | yes | 2 | no | no | yes | yes | no | no | no |  | 23 |  |
| Tran-Dinh (118) | 2018 | France | 01.10.2013 | 31.10.2015 | retrospective cohort study | no | 1 | no | no | no | no | no | yes | no | 25 |  |  |
| Voor in't holt (119) | 2018 | Netherlands | 01.08.2003 | 30.04.2015 | retrospective case-control study | yes | 1 | no | no | no | yes | no | no | no | 20 |  |  |
| Wendel (120) | 2015 | Germany | 2002 | 2014 | outbreak | yes | 1 | no | no | no | no | no | yes | no |  | 23 |  |
| Willmann (121) | 2015 | Germany | 2009 | 2012 | outbreak | yes | 3 | no | no | yes | yes | no | yes | no |  | 18 |  |
| Yakupogullari (122) | 2008 | Turkey | 1.11.2004 | 31.05.2005 | outbreak | yes | 2 | no | no | no | no | yes | yes | no |  | 17 |  |
| Yapicioglu (123) | 2012 | Turkey | 01.06.2010 | 28.02.2011 | outbreak | yes | 2 | no | no | no | no | yes | yes | no |  | 17 |  |
| Yetkin (124) | 2017 | Turkey | 01.11.2007 | 28.02.2008 | outbreak | yes | 1 | no | no | no | no | no | yes | no |  | 23 |  |
| Zhou (125) | 2016 | China | na | na | prospective cohort study | no | 4 | no | no | yes | yes | yes | yes | no | 15 |  |  |
| Zoltanski (126) | 2011 | USA | na | na | prospective cohort study | no | 1 | no | no | no | yes | no | no | no | 15 |  |  |

Abbreviations: OI = outbreak investigations, HCW = healthcare workers, STROBE = Strengthening the reporting of observational studies in epidemiology, ORION = guidelines for transparent reporting of Outbreak Reports and Intervention studies of Nosocomial infection, CARE = Consensus-based Clinical Case Reporting Guideline.

References Additional Table2:

1. Achour W, Abbassi MS, Cherif A, Jabnoun S, Khrouf N, Ben Hassen A. Nosocomial respiratory infection due to an imipenem-resistant *Pseudomonas aeruginosa* O:12 strain in a Tunis's neonatal intensive care unit. Pathol Biol. 2006;54(10):596-9.

2. Adachi JA, Perego C, Graviss L, Dvorak T, Hachem R, Chemaly RF, et al. The role of interventional molecular epidemiology in controlling clonal clusters of multidrug resistant *Pseudomonas aeruginosa* in critically ill cancer patients. Am J Infect Control. 2009;37(6):442-6.

3. Aditi, Shariff M, Chhabra SK, Rahman MU. Similar virulence properties of infection and colonization associated *Pseudomonas aeruginosa*. J Med Microbiol. 2017;66(10):1489-98.

4. Agodi A, Barchitta M, Cipresso R, Giaquinta L, Romeo MA, Denaro C. *Pseudomonas aeruginosa* carriage, colonization, and infection in ICU patients. Intensive Care Med. 2007;33(7):1155-61.

5. Aguilera-Saez J, Andreu-Sola V, Larrosa Escartin N, Rodriguez Garrido V, Armadans Gil L, Sanchez Garcia JM, et al. Extensively drug-resistant *Pseudomonas aeruginosa* outbreak in a burn unit: management and solutions. Ann Burns Fire Disasters. 2019;32(1):47-55.

6. Alipour N, Karagoz A, Taner A, Gaeini N, Alipour N, Zeytin H, et al. Outbreak of Hospital Infection from Biofilm-embedded Pan Drug-resistant *Pseudomonas aeroginosa*, Due to a Contaminated Bronchoscope. J Prev Med (Wilmington). 2017;2(2).

7. Ambrogi V, Cavalié L, Mantion B, Ghiglia MJ, Cointault O, Dubois D, et al. Transmission of metallo-β-lactamase-producing *Pseudomonas aeruginosa* in a nephrology-transplant intensive care unit with potential link to the environment. J Hosp Infect. 2016;92(1):27-9.

8. Araoka H, Kimura M, Abe M, Takahashi N, Yoneyama A. Appropriate Sampling Sites for the Surveillance of Multidrug-Resistant *Pseudomonas aeruginosa* Colonization. Japanese Journal of Infectious Diseases. 2014;67(2):118-9.

9. Aubron C, Poirel L, Fortineau N, Nicolas P, Collet L, Nordmann P. Nosocomial spread of *Pseudomonas aeruginosa* isolates expressing the metallo-β-lactamase VIM-2 in a hematology unit of a French hospital. Microb Drug Resist. 2005;11(3):254-9.

10. Azimi L, Alaghehbandan R, Asadian M, Alinejad F, Lari AR. Multi-drug resistant *Pseudomonas aeruginosa* and *Klebsiella pneumoniae* circulation in a burn hospital, Tehran, Iran. GMS Hyg Infect Control. 2019;14:Doc01.

11. Bertrand X, Bailly P, Blasco G, Balvay P, Boillot A, Talon D. Large outbreak in a surgical intensive care unit of colonization or infection with P*seudomonas aeruginosa* that overexpressed an active efflux pump. Clin Infect Dis. 2000;31(4):E9-E14.

12. Bertrand X, Thouverez M, Patry C, Balvay P, Talon D. *Pseudomonas aeruginosa*: Antibiotic susceptibility and genotypic characterization of strains isolated in the intensive care unit. Clin Microbiol Infect. 2001;7(12):706-8.

13. Bilavsky E, Pfeffer I, Tarabeia J, Schechner V, Abu-Hanna J, Grisaru-Soen G, et al. Outbreak of multidrug-resistant *Pseudomonas aeruginosa* infection following urodynamic studies traced to contaminated transducer. J Hosp Infect. 2013;83(4):344-6.

14. Bingen E, Bonacorsi S, Rohrlich P, Duval M, Lhopital S, Brahimi N, et al. Molecular epidemiology provides evidence of genotypic heterogeneity of multidrug-resistant *Pseudomonas aeruginosa* serotype O:12 outbreak isolates from a pediatric hospital. J CLIN MICROBIOL. 1996;34(12):3226-9.

15. Biswal I, Arora BS, Kasana D, Neetushree. Incidence of multidrug resistant *Pseudomonas aeruginosa* isolated from burn patients and environment of teaching institution. J Clin Diagn Res. 2014;8(5):26-9.

16. Boutiba-Ben Boubake I, Boukadida J, Triki O, Hannachi N, Ben Redjeb S. Outbreak of nosocomial urinary tract infections due to a multidrug resistant *Pseudomonas aeruginosa*. Pathol Biol. 2003;51(3):147-50.

17. Bradbury RS, Champion AC, Reid DW. Epidemiology of *Pseudomonas aeruginosa* in a tertiary referral teaching hospital. J Hosp Infect. 2009;73(2):151-6.

18. Breathnach AS, Cubbon MD, Karunaharan RN, Pope CF, Planche TD. Multidrug-resistant *Pseudomonas aeruginosa* outbreaks in two hospitals: Association with contaminated hospital waste-water systems. J Hosp Infect. 2012;82(1):19-24.

19. Buhl M, Kastle C, Geyer A, Autenrieth IB, Peter S, Willmann M. Molecular Evolution of Extensively Drug-Resistant (XDR) *Pseudomonas aeruginosa* Strains From Patients and Hospital Environment in a Prolonged Outbreak. Front Microbiol. 2019;10:1742.

20. Bukholm G, Tannæs T, Kjelsberg ABB, Smith-Erichsen N. An outbreak of multidrug-resistant *Pseudomonas aeruginosa* associated with increased risk of patient death in an intensive care unit. Infect Control Hosp Epidemiol. 2002;23(8):441-6.

21. Carattoli AF, D.; Galetti, R.; Garcia-Fernandez, A.; Nardi, G.; Orazi, D.; Capone, A.; Majolino, I.; Proia, A.; Mariani, B.; Parisi, G.; Morrone, A.; Petrosillo, N. Isolation of NDM-1-producing *Pseudomonas aeruginosa* sequence type ST235 from a stem cell transplant patient in Italy, May 2013. Eurosurveillance. 2013;18(46):20633.

22. Catho G, Martischang R, Boroli F, Chraiti MN, Martin Y, Koyluk Tomsuk Z, et al. Outbreak of *Pseudomonas aeruginosa* producing VIM carbapenemase in an intensive care unit and its termination by implementation of waterless patient care. Crit Care. 2021;25(1):301.

23. Cezário RC, Duarte De Morais L, Ferreira JC, Costa-Pinto RM, Da Costa Darini AL, Gontijo-Filho PP. Nosocomial outbreak by imipenem-resistant metallo-β-lactamase- producing *Pseudomonas aeruginosa* in an adult intensive care unit in a Brazilian teaching hospital. Enferm Infecc Microbiol Clin. 2009;27(5):269-74.

24. Chaves L, Tomich LM, Salomão M, Leite GC, Ramos J, Martins RR, et al. High mortality of bloodstream infection outbreak caused by carbapenem-resistant *P. aeruginosa* producing SPM-1 in a bone marrow transplant unit. J Med Microbiol. 2017;66(12):1722-9.

25. Cholley P, Gbaguidi-Haore H, Bertrand X, Thouverez M, Plésiat P, Hocquet D, et al. Molecular epidemiology of multidrug-resistant *Pseudomonas aeruginosa* in a French university hospital. J Hosp Infect. 2010;76(4):316-9.

26. Ciofi Degli Atti M, Bernaschi P, Carletti M, Luzzi I, García-Fernández A, Bertaina A, et al. An outbreak of extremely drug-resistant *Pseudomonas aeruginosa* in a tertiary care pediatric hospital in Italy. BMC Infect Dis. 2014;14(1):1-8.

27. Corona-Nakamura AL, Miranda-Novales MG, Leaños-Miranda B, Portillo-Gómez L, Hernández-Chávez A, Anthor-Rendón J, et al. Epidemiologic study of *Pseudomonas aeruginosa* in critical patients and reservoirs. Arch Med Res. 2001;32(3):238-42.

28. Cortes JA, Cuervo SI, Urdaneta AM, Potdevin G, Arroyo P, Bermúdez D, et al. Identifying and controlling a multiresistant *Pseudomonas aeruginosa* outbreak in a latin-american cancer centre and its associated risk factors. Braz J Infect Dis. 2009;13(2):99-103.

29. Corvec S, Poirel L, Espaze E, Giraudeau C, Drugeon H, Nordmann P. Long-term evolution of a nosocomial outbreak of *Pseudomonas aeruginosa* producing VIM-2 metallo-enzyme. J Hosp Infect. 2008;68(1):73-82.

30. Crespo MP, Woodford N, Sinclair A, Kaufmann ME, Turton J, Glover J, et al. Outbreak of carbapenem-resistant *Pseudomonas aeruginosa* producing VIM-8, a novel metallo-β-lactamase, in a tertiary care center in Cali, Colombia. J Clin Microbiol. 2004;42(11):5094-101.

31. Crivaro V, Di Popolo A, Caprio A, Lambiase A, Di Resta M, Borriello T, et al. *Pseudomonas aeruginosa* in a neonatal intensive care unit: Molecular epidemiology and infection control measures. BMC Infect Dis. 2009;9.

32. DalBen MF, Basso M, Garcia CP, Figueiredo Costa S, Maria Toscano C, Robert Jarvis W, et al. Colonization pressure as a risk factor for colonization by multiresistant Acinetobacter spp and carbapenem-resistant *Pseudomonas aeruginosa* in an intensive care unit. Clinics. 2013;68(8):1128-33.

33. de Jonge E, de Boer MGJ, van Essen EHR, Dogterom-Ballering HCM, Veldkamp KE. Effects of a disinfection device on colonization of sink drains and patients during a prolonged outbreak of multidrug-resistant *Pseudomonas aeruginosa* in an intensive care unit. J Hosp Infect. 2019;102(1):70-4.

34. Decraene V, Ghebrehewet S, Dardamissis E, Huyton R, Mortimer K, Wilkinson D, et al. An outbreak of multidrug-resistant *Pseudomonas aeruginosa* in a burns service in the North of England: challenges of infection prevention and control in a complex setting. J Hosp Infect. 2018;100(4):e239-e45.

35. Deplano A, Denis O, Poirel L, Hocquet D, Nonhoff C, Byl B, et al. Molecular characterization of an epidemic clone of panantibiotic-resistant *Pseudomonas aeruginosa*. J Clin Microbiol. 2005;43(3):1198-204.

36. Döring G, Jansen S, Noll H, Grupp H, Frank F, Botzenhart K, et al. Distribution and transmission of *Pseudomonas aeruginos*a and *Burkholderia cepacia* in a hospital ward. PEDIATR PULMONOL. 1996;21(2):90-100.

37. Dubois V, Arpin C, Melon M, Melon B, Andre C, Frigo C, et al. Nosocomial outbreak due to a multiresistant strain of *Pseudomonas aeruginosa* P12: efficacy of cefepime-amikacin therapy and analysis of beta-lactam resistance. J Clin Microbiol. 2001;39(6):2072-8.

38. Dunne EM, Hylsky D, Peterson E, Voermans R, Ward A, Turner K, et al. A cluster of *Achromobacter xylosoxidans* led to identification of *Pseudomonas aeruginosa* and *Serratia marcescens* contamination at a long-term-care facility. Am J Infect Control. 2021;49(10):1331-3.

39. Dwivedi M, Mishra A, Singh RK, Azim AK, Baronia A, Prasad KN. Nosocomial cross-transmission of *Pseudomonas aeruginosa* between patients in a tertiary intensive care unit. Indian J Pathol Microbiol. 2009;52(4):509-13.

40. Elias J, Schoen C, Heinze G, Valenza G, Gerharz E, Riedmiller H, et al. Nosocomial outbreak of VIM-2 metallo-β-lactamase-producing *Pseudomonas aeruginosa* associated with retrograde urography. Clin Microbiol Infect. 2010;16(9):1494-500.

41. Fernández-Cuenca F, López-Cerero L, Cabot G, Oliver A, López-Méndez J, Recacha E, et al. Nosocomial outbreak linked to a flexible gastrointestinal endoscope contaminated with an amikacin-resistant ST17 clone of *Pseudomonas aeruginosa*. Eur J Clin Microbiol Infect Dis. 2020.

42. Ferreira ACB, Gobara S, Costa SF, Sauaia N, Mamizuka EM, Van Der Heijden IM, et al. Emergence of resistance in P*seudomonas aeruginosa* and Acinetobacter species after the use of antimicrobials for burned patients. Infect Control Hosp Epidemiol. 2004;25(10):868-72.

43. Flateau C, Janvier F, Delacour H, Males S, Ficko C, Andriamanantena D, Jeannot K, Merens A, Rapp C. Recurrent pyelonephritis due to NDM-1 metallo-beta-lactamase producing *Pseudomonas aeruginosa* in a patient returning from Serbia, France, 2012. Euro Surveill. 2012 Nov 8;17(45):20311. PMID: 23153474.

44. Fraser TG, Reiner S, Malczynski M, Yarnold PR, Warren J, Noskin GA. Multidrug-resistant *Pseudomonas aeruginosa* cholangitis after endoscopic retrograde cholangiopancreatography: Failure of routine endoscope cultures to prevent an outbreak. Infect Control Hosp Epidemiol. 2004;25(10):856-9.

45. Freire MP, Camargo CH, Yamada AY, Nagamori FO, Reusing Junior JO, Spadao F, et al. Critical points and potential pitfalls of outbreak of IMP-1-producing carbapenem-resistant *Pseudomonas aeruginosa* among kidney transplant recipients: a case-control study. J Hosp Infect. 2021;115:83-92.

46. Furtado GHC, Martins ST, Oliveira Machado AM, Wey SB, Servolo Medeiros EA. Clinical culture surveillance of carbapenem-resistant *Pseudomonas aeruginosa* and Acinetobacter species in a teaching hospital in São Paulo, Brazil: A 7-year study. Infect Control Hosp Epidemiol. 2006;27(11):1270-3.

47. Galdys AL, Marsh JW, Delgado E, Pasculle AW, Pacey M, Ayres AM, et al. Bronchoscope-associated clusters of multidrug-resistant *Pseudomonas aeruginosa* and carbapenem-resistant *Klebsiella pneumoniae*. Infect Control Hosp Epidemiol. 2018;40(1):40-6.

48. Gbaguidi-Haore H, Varin A, Cholley P, Thouverez M, Hocquet D, Bertrand X. A Bundle of Measures to Control an Outbreak of *Pseudomonas aeruginosa* Associated with P-Trap Contamination. Infect Control Hosp Epidemiol. 2018;39(2):164-9.

49. Geladari A, Karampatakis T, Antachopoulos C, Iosifidis E, Tsiatsiou O, Politi L, et al. Epidemiological surveillance of multidrug-resistant gram-negative bacteria in a solid organ transplantation department. Transplant Infect Dis. 2017;19(3).

50. Gibb AP, Tribuddharat C, Moore RA, Louie TJ, Krulicki W, Livermore DM, et al. Nosocomial outbreak of carbapenem-resistant *Pseudomonas aeruginosa* with a new blaIMP allele, blaIMP-7. Antimicrob Agents Chemother. 2002;46(1):255-8.

51. Gomes MZR, Machado CR, De Souza da Conceição M, Ortega JA, Neves SMFM, Da Silva Lourenço MC, et al. Outbreaks, persistence, and high mortality rates of multiresistant *Pseudomonas aeruginos*a infections in a hospital with AIDS-predominant admissions. Braz J Infect Dis. 2011;15(4):312-22.

52. Goudarzi H, Ghazi M, Khanbabaee G, Fallah F, Kazemi B, Mahmoudi S, et al. Emergence of *Pseudomonas aeruginosa* cross-infection in children with cystic fibrosis attending an Iranian referral pediatric center. Iran J Microbiol. 2012;4(3):124-9.

53. Griffith SJ, Nathan C, Selander RK, Chamberlin W, Gordon S, Kabins S, et al. The epidemiology of *Pseudomonas aeruginosa* in oncology patients in a General Hospital. J INFECT DIS. 1989;160(6):1030-6.

54. Gülay Z, Atay T, Amyes SGB. Clonal spread of imipenem-resistant *Pseudomonas aeruginosa* in the intensive care unit of a Turkish hospital. J Chemother. 2001;13(5):546-54.

55. Harris AD, Johnson JK, Thom KA, Morgan DJ, McGregor JC, Ajao AO, et al. Risk factors for development of intestinal colonization with imipenem-resistant *Pseudomonas aeruginosa* inthe intensive care unit setting. Infect Control Hosp Epidemiol. 2011;32(7):719-22.

56. Hopman J, Meijer C, Kenters N, Coolen JPM, Ghamati MR, Mehtar S, et al. Risk Assessment After a Severe Hospital-Acquired Infection Associated With Carbapenemase-Producing *Pseudomonas aeruginosa*. JAMA Netw Open. 2019;2(2):e187665.

57. Hota S, Hirji Z, Stockton K, Lemieux C, Dedier H, Wolfaardt G, et al. Outbreak of multidrug-resistant *Pseudomonas aeruginosa* colonization and infection secondary to imperfect intensive care unit room design. Infect Control Hosp Epidemiol. 2009;30(1):25-33.

58. Hsueh PR, Teng LJ, Yang PC, Chen YC, Ho SW, Luh KT. Persistence of a multidrug-resistant *Pseudomonas aeruginosa* clone in an intensive care burn unit. J Clin Microbiol. 1998;36(5):1347-51.

59. Hu P, Chen J, Chen Y, Zhou T, Xu X, Pei X. Molecular epidemiology, resistance, and virulence properties of *Pseudomonas aeruginosa* cross-colonization clonal isolates in the non-outbreak setting. Infec Genet Evol. 2017;55:288-96.

60. Inglis TJ, Benson KA, O'Reilly L, Bradbury R, Hodge M, Speers D, et al. Emergence of multi-resistant *Pseudomonas aeruginos*a in a Western Australian hospital. J Hosp Infect. 2010;76(1):60-5.

61. Kanayama A, Kawahara R, Yamagishi T, Goto K, Kobaru Y, Takano M, et al. Successful control of an outbreak of GES-5 extended-spectrum β-lactamase-producing *Pseudomonas aeruginosa* in a long-term care facility in Japan. J Hosp Infect. 2016;93(1):35-41.

62. Karami P, Mohajeri P, Yousefi Mashouf R, Karami M, Yaghoobi MH, Dastan D, et al. Molecular characterization of clinical and environmental *Pseudomonas aeruginosa* isolated in a burn center. Saudi J Biol Sci. 2019;26(7):1731-6.

63. Kateete DP, Nakanjako R, Namugenyi J, Erume J, Joloba ML, Najjuka CF. Carbapenem resistant *Pseudomonas aeruginosa* and *Acinetobacter baumannii* at Mulago Hospital in Kampala, Uganda (2007-2009). Springerplus. 2016;5(1):1308.

64. Kikuchi T, Nagashima G, Taguchi K, Kuraishi H, Nemoto H, Yamanaka M, et al. Contaminated oral intubation equipment associated with an outbreak of carbapenem-resistant pseudomonas in an intensive care unit. J Hosp Infect. 2007;65(1):54-7.

65. Knoester M, de Boer MGJ, Maarleveld JJ, Claas ECJ, Bernards AT, de Jonge E, et al. An integrated approach to control a prolonged outbreak of multidrug-resistant *Pseudomonas aeruginosa* in an intensive care unit. Clin Microbiol Infect. 2014;20(4):O207-O15.

66. Kohlenberg A, Weitzel-Kage D, van der Linden P, Sohr D, Vögeler S, Kola A, et al. Outbreak of carbapenem-resistant *Pseudomonas aeruginosa* infection in a surgical intensive care unit. J Hosp Infect. 2010;74(4):350-7.

67. Kossow A, Kampmeier S, Willems S, Berdel WE, Groll AH, Burckhardt B, et al. Control of multidrug-resistant *Pseudomonas aeruginosa* in allogeneic hematopoietic stem cell transplant recipients by a novel bundle including remodeling of sanitary and water supply systems. Clin Infect Dis. 2017;65(6):935-42.

68. Kouda S, Fujiue Y, Watanabe Y, Ohara M, Kayama S, Kato F, et al. Sporadic isolations of a multi-drug resistant *Pseudomonas aeruginosa* clone during a 14-month epidemic in a general hospital in Hiroshima. Infection. 2011;39(3):247-53.

69. Kousouli E, Zarkotou O, Politi L, Polimeri K, Vrioni G, Themeli-Digalaki K, et al. Infection control interventions affected by resource shortages: impact on the incidence of bacteremias caused by carbapenem-resistant pathogens. Eur J Clin Microbiol Infect Dis. 2018;37(1):43-50.

70. Kovaleva J, Meessen NEL, Peters FTM, Been MH, Arends JP, Borgers RP, et al. Is bacteriologic surveillance in endoscope reprocessing stringent enough? Endoscopy. 2009;41(10):913-6.

71. Kristie J, Smith G, Lee MS, Venezia RA, Colin Stine O, Nataro JP, et al. The Role of Patient-to-Patient Transmission in the Acquisition of Imipenem-Resistant *Pseudomonas aeruginosa* Colonization in the Intensive Care Unit. J Infect Dis. 2009;200(6):900-5.

72. Kumarage J, Khonyongwa K, Khan A, Desai N, Hoffman P, Taori SK. Transmission of multi-drug resistant *Pseudomonas aeruginosa* between two flexible ureteroscopes and an outbreak of urinary tract infection: the fragility of endoscope decontamination. J Hosp Infect. 2019;102(1):89-94.

73. Ling ML, How KB. *Pseudomonas aeruginosa* outbreak linked to sink drainage design. Healthc Infect. 2013;18(4):143-6.

74. Liu L, Liu B, Li Y, Zhang W. Successful control of resistance in *Pseudomonas aeruginosa* using antibiotic stewardship and infection control programs at a Chinese university hospital: A 6-year prospective study. Infect Drug Resist. 2018;11:637-46.

75. Loconsole D, Accogli M, Monaco M, Del Grosso M, De Robertis AL, Morea A, et al. First detection of autochthonous extensively drug-resistant NDM-1 *Pseudomonas aeruginosa* ST235 from a patient with bloodstream infection in Italy, October 2019. Antimicrob Resist Infect Control. 2020;9(1).

76. Lolans K, Queenan AM, Bush K, Sahud A, Quinn JP. First nosocomial outbreak of *Pseudomonas aeruginosa* producing an integron-borne metallo-β-lactamase (VIM-2) in the United States. Antimicrob Agents Chemother. 2005;49(8):3538-40.

77. Lyytikäinen O, Golovanova V, Kolho E, Ruutu P, Sivonen A, Tiittanen L, et al. Outbreak caused by tobramycin-resistant *Pseudomonas aeruginosa* in a bone marrow transplantation unit. Scand J Infect Dis. 2001;33(6):445-9.

78. Machida H, Seki M, Yoshioka N, Yabuno K, Miyawaki K, Yoshida H, et al. Correlation between outbreaks of multidrug-resistant *Pseudomonas aeruginos*a infection and use of bronchoscopes suggested by epidemiological analysis. Biol Pharm Bull. 2014;37(1):26-30.

79. Mahmoud MF, Fathy FM, Gohar MK, Awad WM, Soliman MH. Genotyping of *Pseudomonas aeruginosa* strains isolated from surgical site infected patients by RAPD-PCR. Syst Rev Pharm. 2020;11(12):1998-2005.

80. Mammina C, Di Carlo P, Cipolla D, Casuccio A, Tantillo M, Plano MRA, et al. Nosocomial colonization due to imipenem-resistant *Pseudomonas aeruginosa* epidemiologically linked to breast milk feeding in a neonatal intensive care unit. Acta Pharmacol Sin. 2008;29(12):1486-92.

81. Matar GM, Chaar MH, Araj GF, Srour Z, Jamaleddine G, Hadi U. Detection of a highly prevalent and potentially virulent strain of *Pseudomonas aeruginosa* from nosocomial infections in a medical center. BMC Microbiol. 2005;5.

82. Mayr A, Hinterberger G, Lorenz IH, Kreidl P, Mutschlechner W, Lass-Flörl C. Nosocomial outbreak of extensively drug-resistant *Pseudomonas aeruginosa* associated with aromatherapy. Am J Infect Control. 2017;45(4):453-5.

83. Mendes ET, Salomão MC, Tomichi LM, Oliveira MS, Graça M, Rossi F, et al. Effectiveness of surveillance cultures for high priority multidrug-resistant bacteria in hematopoietic stem cell transplant units. Rev Inst Med Trop Sao Paulo. 2021;63.

84. Mentzelopoulos SD, Pratikaki M, Platsouka E, Kraniotaki H, Zervakis D, Koutsoukou A, et al. Prolonged use of carbapenems and colistin predisposes to ventilator-associated pneumonia by pandrug-resistant *Pseudomonas aeruginosa*. Intensive Care Med. 2007;33(9):1524-32.

85. Milan O, Debroize L, Bertrand X, Plesiat P, Valentin AS, Quentin R, et al. Difficult-to-detect carbapenem-resistant IMP13-producing *P. aeruginosa*: Experience feedback concerning a cluster of urinary tract infections at a surgical clinic in France. Antimicrob Resist Infect Control. 2013;2(1).

86. Miranda G, Leanos B, Marquez L, Valenzuela A, Silva J, Carrillo B, et al. Molecular epidemiology of a multiresistant *Pseudomonas aeruginosa* outbreak in a paediatric intensive care unit. Scand J Infect Dis. 2001;33(10):738-43.

87. Moremi N, Claus H, Vogel U, Mshana SE. Surveillance of surgical site infections by *Pseudomonas aeruginosa* and strain characterization in Tanzanian hospitals does not provide proof for a role of hospital water plumbing systems in transmission. Antimicrob Resist Infect Control. 2017;6(1).

88. Mudau M, Jacobson R, Minenza N, Kuonza L, Morris V, Engelbrecht H, et al. Outbreak of Multi-Drug Resistant *Pseudomonas aeruginosa* Bloodstream Infection in the Haematology Unit of a South African Academic Hospital. Plos One. 2013;8(3).

89. Muthu SE, Aberna RA, Mohan V, Premalatha G, Srinivasan RS, Thyagarajan SP, et al. Phenotypes of isolates o*f Pseudomonas aeruginosa* in a diabetes care center. Arch Med Res. 2006;37(1):95-101.

90. Nagao M, Iinuma Y, Igawa J, Saito T, Yamashita K, Kondo T, et al. Control of an outbreak of carbapenem-resistant *Pseudomonas aeruginosa* in a haemato-oncology unit. J Hosp Infect. 2011;79(1):49-53.

91. Orsi GB. Lack of association between clinical and environmental isolates of *Pseudomonas aeruginosa* in hospital wards. J HOSP INFECT. 1994;27(1):49-60.

92. Ozer B, Tatman-Otkun M, Memis D, Otkun M. Characteristics of *Pseudomonas aeruginosa* isolates from intensive care unit. Cent Eur J Med. 2009;4(2):156-63.

93. Panzig B, Schröder G, Pitten FA, Gründling M. A large outbreak of multiresistant *Pseudomonas aeruginosa* strains in north-eastern Germany. J Antimicrob Chemother. 1999;43(3):415-8.

94. Parcell BJ, Oravcova K, Pinheiro M, Holden MTG, Phillips G, Turton JF, et al. *Pseudomonas aeruginosa* intensive care unit outbreak: winnowing of transmissions with molecular and genomic typing. J Hosp Infect. 2018;98(3):282-8.

95. Pelegrin AC, Saharman YR, Griffon A, Palmieri M, Mirande C, Karuniawati A, et al. High-risk international clones of carbapenem-nonsusceptible *Pseudomonas aeruginosa* endemic to Indonesian intensive care units: Impact of a multifaceted infection control intervention analyzed at the genomic level. mBio. 2019;10(6).

96. Peña C, Dominguez MA, Pujol M, Verdaguer R, Gudiol F, Ariza J. An outbreak of carbapenem-resistant *Pseudomonas aeruginosa* in a urology ward. Clin Microbiol Infect. 2003;9(9):938-43.

97. Peña C, Suarez C, Tubau F, Gutierrez O, Domínguez A, Oliver A, et al. Nosocomial spread of *Pseudomonas aeruginosa* producing the metallo-β-lactamase VIM-2 in a Spanish hospital: Clinical and epidemiological implications. Clin Microbiol Infect. 2007;13(10):1026-9.

98. Pirnay JP, De Vos D, Cochez C, Bilocq F, Pirson J, Struelens M, et al. Molecular epidemiology of *Pseudomonas aeruginosa* colonization in a burn unit: Persistence of a multidrug-resistant clone and a silver sulfadiazine-resistant clone. J Clin Microbiol. 2003;41(3):1192-202.

99. Pitten FA, Panzig B, Schröder G, Tietze K, Kramer A. Transmission of a multiresistant *Pseudomonas aeruginosa* strain at a German University Hospital. J Hosp Infect. 2001;47(2):125-30.

100. Prashanth K, Singh SK, Kanungo R, Sharma S, Shashikala P, Joshi S, et al. Correlations between genotyping and antibiograms of clinical isolates of *Pseudomonas aeruginosa* from three different south Indian hospitals. Indian J Med Microbiol. 2010;28(2):130-7.

101. Quick J, Cumley N, Wearn CM, Niebel M, Constantinidou C, Thomas CM, et al. Seeking the source of *Pseudomonas aeruginosa* infections in a recently opened hospital: An observational study using whole-genome sequencing. BMJ Open. 2014;4(11).

102. Ramírez DG, Nicola F, Zarate S, Relloso S, Smayevsky J, Arduino S. Emergence of *Pseudomonas aeruginosa* with KPC-type carbapenemase in a teaching hospital: An 8-year study. J Med Microbiol. 2013;62(PART10):1565-70.

103. Richard P, Le Floch R, Chamoux C, Pannier M, Espaze E, Richet H. *Pseudomonas aeruginosa* outbreak in a burn unit: Role of antimicrobials in the emergence of multiply resistant strains. J INFECT DIS. 1994;170(2):377-83.

104. Ruiz L, Domínguez MA, Ruiz N, Viñas M. Relationship between clinical and environmental isolates of *Pseudomonas aeruginosa* in a hospital setting. Arch Med Res. 2004;35(3):251-7.

105. Sader HS, Pignatari AC, Leme IL, Burattini MN, Tancresi R, Hollis RJ, et al. Epidemiologic typing of multiply drug-resistant *Pseudomonas aeruginosa* isolated from an outbreak in an intensive care unit. DIAGN MICROBIOL INFECT DIS. 1993;17(1):13-8.

106. Saharman YR, Pelegrin AC, Karuniawati A, Sedono R, Aditianingsih D, Goessens WHF, et al. Epidemiology and characterisation of carbapenem-non-susceptible *Pseudomonas aeruginosa* in a large intensive care unit in Jakarta, Indonesia. Int J Antimicrob Agents. 2019;54(5):655-60.

107. Salauze B, Badaoui H, Gholizadeh Y, Bure-Rossier A. Outbreak of nosocomial infections due to serotype O11 multiresistant *Pseudomonas aeruginosa*. MED MAL INFECT. 1997;27(SPEC. ISS. JUNE):658-62.

108. Salimi H, Yakhchali B, Owlia P, Lari AR. Molecular epidemiology and drug susceptibility of *Pseudomonas aeruginosa* strains isolated from burn patients. Lab Med. 2010;41(9):540-4.

109. Salm F, Deja M, Gastmeier P, Kola A, Hansen S, Behnke M, et al. Prolonged outbreak of clonal MDR *Pseudomonas aeruginosa* on an intensive care unit: Contaminated sinks and contamination of ultra-filtrate bags as possible route of transmission? Antimicrob Resist Infect Control. 2016;5(1).

110. Schäfer E, Malecki M, Tellez-Castillo CJ, Pfennigwerth N, Marlinghaus L, Higgins PG, et al. Molecular surveillance of carbapenemase-producing *Pseudomonas aeruginosa* at three medical centres in Cologne, Germany. Antimicrob Resist Infect Control. 2019;8(1).

111. Seki M, Machida N, Yamagishi Y, Yoshida H, Tomono K. Nosocomial outbreak of multidrug-resistant *Pseudomonas aeruginosa* caused by damaged transesophageal echocardiogram probe used in cardiovascular surgical operations. J Infect Chemother. 2013;19(4):677-81.

112. Sekiguchi JI, Teruya K, Horii K, Kuroda E, Konosaki H, Mizuguchi Y, et al. Molecular epidemiology of outbreaks and containment of drug-resistant *Pseudomonas aeruginosa* in a Tokyo hospital. J Infect Chemother. 2007;13(6):418-22.

113. Shigemura K, Takase R, Osawa K, Takaba K, Nomi M, Fujisawa M, et al. Emergence and prevention measures for multidrug resistant *Pseudomonas aeruginosa* in catheter-associated urinary tract infection in spinal cord injury patients. Spinal Cord. 2015;53(1):70-4.

114. Snyder LA, Loman NJ, Faraj LA, Levi K, Weinstock G, Boswell TC, et al. Epidemiological investigation of *Pseudomonas aeruginosa* isolates from a six-year-long hospital outbreak using high-throughput whole genome sequencing. Eurosurveillance. 2013;18(42).

115. Sorin M, Segal-Maurer S, Mariano N, Urban C, Combest A, Rahal JJ. Nosocomial transmission of imipenem-resistant *Pseudomonas aeruginosa* following bronchoscopy associated with improper connection to the steris system I processor. Infect Control Hosp Epidemiol. 2001;22(7):409-13.

116. Suarez C, Peña C, Arch O, Dominguez MA, Tubau F, Juan C, et al. A large sustained endemic outbreak of multiresistant *Pseudomonas aeruginosa*: A new epidemiological scenario for nosocomial acquisition. BMC Infect Dis. 2011;11.

117. Talon D, Capellier G, Boillot A, Michel-Briand Y. Use of pulsed-field gel electrophoresis as an epidemiologic tool during an outbreak of *Pseudomonas aeruginosa* lung infections in an intensive care unit. Intensive Care Med. 1995;21(12):996-1002.

118. Tran-Dinh A, Neulier C, Amara M, Nebot N, Troché G, Breton N, et al. Impact of intensive care unit relocation and role of tap water on an outbreak of *Pseudomonas aeruginosa* expressing OprD-mediated resistance to imipenem. J Hosp Infect. 2018;100(3):e105-e14.

119. Voor in 't holt AF, Severin JA, Hagenaars MBH, de Goeij I, Gommers D, Vos MC. VIM-positive *Pseudomonas aeruginosa* in a large tertiary care hospital: Matched case-control studies and a network analysis. Antimicrob Resist Infect Control. 2018;7(1).

120. Wendel AF, Kolbe-Busch S, Ressina S, Schulze-Röbbecke R, Kindgen-Milles D, Lorenz C, et al. Detection and termination of an extended low-frequency hospital outbreak of GIM-1-producing *Pseudomonas aeruginosa* ST111 in Germany. Am J Infect Control. 2015;43(6):635-9.

121. Willmann M, Bezdan D, Zapata L, Susak H, Vogel W, Schröppel K, et al. Analysis of a long-term outbreak of XDR *Pseudomonas aeruginosa*: A molecular epidemiological study. J Antimicrob Chemother. 2014;70(5):1322-30.

122. Yakupogullari Y, Otlu B, Dogukan M, Gursoy C, Korkmaz E, Kizirgil A, et al. Investigation of a nosocomial outbreak by alginate-producing pan-antibiotic-resistant *Pseudomonas aeruginosa*. Am J Infect Control. 2008;36(10):e13-e8.

123. Yapicioglu H, Gokmen TG, Yildizdas D, Koksal F, Ozlu F, Kale-Cekinmez E, et al. *Pseudomonas aeruginosa* infections due to electronic faucets in a neonatal intensive care unit. J Paediatr Child Health. 2012;48(5):430-4.

124. Yetkin F, Ersoy Y, Kuzucu C, Otlu B, Parmaksiz N, Seckin Y. An outbreak associated with multidrug-resistant *Pseudomonas aeruginosa* contamination of duodenoscopes and an automated endoscope reprocessor. Biomed Res. 2017;28(13):6064-70.

125. Zhou Z, Hu B, Gao X, Bao R, Chen M, Li H. Sources of sporadic *Pseudomonas aeruginosa* colonizations/infections in surgical ICUs: Association with contaminated sink trap. J Infect Chemother. 2016;22(7):450-5.

126. Zoltanski J, Dul M, O'Riordan MA, Blumer J, Toltzis P. Low frequency of endemic patient-to-patient transmission of antibiotic-resistant gram-negative bacilli in a pediatric intensive care unit. Infect Control Hosp Epidemiol. 2011;32(9):915-7.

**Additional Table 3.** Outbreak investigations after detection of CRPA stratified by hospital setting.

|  | Tertiary care center  (N = 93) | Secondary care center  (N = 2) | Primary care center  (N = 2) | LTCF  ( N = 2) | Rehabilitation center  (N = 1) | Not available (N = 26) |
| --- | --- | --- | --- | --- | --- | --- |
| Median number of components (range) | **2.0 (1-5)** | **2.5 (2-3)** | **3 (2-4)** | **2.5 (1-4)** | **1 (1)** | **2.0 (1-4)** |
| 1) Identifying contact patients (%) | 14 (15.1) | 1 (50) | 2 (100) | 1 (50.0) | 1 (100) | 6 (23.1) |
| 2) Screening of contact patients (%) | 11 (11.8)^1^ | 1 (50) | 2 (100) | 1 (50.0) | 1 (100) | 6 (23.1) |
| 3) Screening on admission (%) | 27 (29.0)^1^ | 2 (100) | 1 (50) | 0 | 1 (100) | 6 (23.1) |
| 4) Screening during hospitalization (%) | 40 (43.0) | 1 (50) | 1 (50) | 1 (50) | 1 (100) | 7 (26.9) |
| 5) Screening of HCW (%) | 29 (31.2) | 2 (100) | 2 (100) | 0 | 1 (100) | 6 (23.1) |
| 6) Screening of the environment (%)  Dry environment (%)  Wet environment (%) | 75 (80.6)  45 (48.4)^3^  74 (79.6)^1^ | 2 (100)  2 (100)  2 (100) | 2 (100)  1 (50)  2 (100) | 2 (100)  1 (50)^2^  2 (100) | 1 (100)  1 (100)  1 (100) | 19 (73.1)^2^  6 (23.1)^4^  13 (50)^5^ |
| 7) Other (%) | 1 (5.4) | 0 | 1 (50) | 0 | 0 | 1 (3.8) |

Abbreviations: HCW = healthcare workers, LTCF = long-term care facility. ^1^Information from two studies missing, ^2^information from one study missing, ^3^information from four studies missing, ^4^information from seven studies missing, ^5^information from five studies missing.

**Additional Table 4.** Description of the screening of HCW in 37 studies (29.4%).

| Study | Screening site(s) | Number of HCW screened | Number of HCW screened positive for CRPA | Number of HCW screened positive for CRPA identical to the patient’s isolate |
| --- | --- | --- | --- | --- |
| Achour et al. 2006 | hands | not described | 1 | 0 |
| Aditi et al. 2017 | not described | not described | not described | not described |
| Alipour et al 2017 | hands | 5 | not described | not described |
| Bertrand et al. 2000 | hands | 110 | not described | 2 |
| Biswal et al. 2014 | hands, nose, throat, | 51 | 0 | 0 |
| Carattoli et al. 2013 | hands, additional sites not described | not described | 0 | 0 |
| Cezario et al. 2009 | hands | 23 | 0 | 0 |
| Chaves et al. 2017 | hands | not described | 0 | 0 |
| Corona-Nakamura et al 2001 | hands | 47 | 3 | 3 |
| Crespo et al. 2004 | hands | 10 | 0 | 0 |
| Crivaro et al. 2009 | hands | not described | not described | 1 |
| Deplano et al. 2005 | hands | 10 | not described | 1 |
| Döring et al. 1996 | hands | 79 | 11 | 1 |
| Dubois et al. 2001 | hands | 8 | 0 | 0 |
| Dwivedi et al. 2009 | hands | 120 | 34 | 27 |
| Gomes et al. 2011 | hands | not described | 0 | 0 |
| Griffith et al. 1989 | hands | 63 | 0 | 0 |
| Gülay et al. 2001 | hands | 50 | 0 | 0 |
| Hsueh et al. 1998 | hands, nose | not described | 0 | 0 |
| Inglis et al. 2010 | hands | 28 | 0 | 0 |
| Liu et al. 2018 | hands | not described | not described | not described |
| Loconsole et al. 2020 | not described | not described | not described | not described |
| Mahmoud et al. 2020 | hands | 30 | 3 | not described |
| Mayr et al. 2017 | hands, nose, throat | not described | 0 | 0 |
| Mentzelopoulos et al. 2007 | hands | not described | 0 | 0 |
| Miranda et al. 2001 | hands | 29 | 0 | 0 |
| Mudau et al. 2013 | stool | 26 | 0 | 0 |
| Ozer et al. 2009 | hands, nose | not described | 3 | 1 |
| Panzig et al. 1999 | hands, nose, throat | not described | 0 | 0 |
| Pelegrin et al. 2019 | not described | not described | not described | not described |
| Pirnay et al. 2003 | hands | not described | 0 | 0 |
| Pitten et al. 2001 | hands | 27 | 0 | 0 |
| Sader et al. 1993 | hands | not described | 0 | 0 |
| Saharman et al. 2019 | throat, rectal | 25 | 0 | 0 |
| Yakupogullari et al. 2008 | hands | 40 | 3 | 3 |
| Yapicioglu et al. 2008 | hands | not described | 0 | 0 |
| Zhou et al. 2016 | hands | 23 | 0 | 0 |

Abbreviations: HCW = healthcare workers, CRPA = carbapenem-resistant *Pseudomonas aeruginosa*.

**Additional Table 5.** Quality assessments of the included studies by study type.

| Quality Threshold scores | | Strobe^1^ (N=55) | Orion^2^ (N=68) | Care^3^ (N=3) |
| --- | --- | --- | --- | --- |
| Low (%) | Strobe 0-11 | 15 (27.3) |  |  |
|  | Orion 0-17 |  | 38 (55.9) |  |
|  | Care 0-10 |  |  | 0 (0) |
| Moderate (%) | Strobe 12-22  Orion 18-34  Care 11-20 | 35 (63.6) | 30 (44.1) | 3 (100) |
| High (%) | Strobe 23-33  Orion 35-52  Care 21-30 | 5 (9.1) | 0 (0) | 0 (0) |

**^1^** von Elm E. ADG, Pocock S.J., Gotysche P.C., Vandenbroucke J.P., for the STROBE Initiative. Strengthening the reporting of observational studies in epidemiology (STROBE) statement: guidelines for reporting observational studies. BMJ **2007**; 335: 806-8. **^2^** Stone SP, Cooper BS, Kibbler CC, et al. The ORION statement: guidelines for transparent reporting of outbreak reports and intervention studies of nosocomial infection. Lancet Infect Dis **2007**; 7: 282-88. **^3^** Riley DS, Barber MS, Kienle GS, et al. CARE guidelines for case reports: explanation and elaboration document. J Clin Epidemiol **2017**; 89: 218-35.

**Additional Document 1.** PRISMA 2020 Checklist

| **Section and Topic** | **Item #** | **Checklist item** | **Location where item is reported** |
| --- | --- | --- | --- |
| **TITLE** | | |  |
| Title | 1 | Identify the report as a systematic review. | Title |
| **ABSTRACT** | | |  |
| Abstract | 2 | See the PRISMA 2020 for Abstracts checklist. | Abstract |
| **INTRODUCTION** | | |  |
| Rationale | 3 | Describe the rationale for the review in the context of existing knowledge. | Introduction |
| Objectives | 4 | Provide an explicit statement of the objective(s) or question(s) the review addresses. | Introduction |
| **METHODS** | | |  |
| Eligibility criteria | 5 | Specify the inclusion and exclusion criteria for the review and how studies were grouped for the syntheses. | Methods/Study selection |
| Information sources | 6 | Specify all databases, registers, websites, organisations, reference lists and other sources searched or consulted to identify studies. Specify the date when each source was last searched or consulted. | Methods/Study selection and Table 1 |
| Search strategy | 7 | Present the full search strategies for all databases, registers and websites, including any filters and limits used. | Supplementary document 2 |
| Selection process | 8 | Specify the methods used to decide whether a study met the inclusion criteria of the review, including how many reviewers screened each record and each report retrieved, whether they worked independently, and if applicable, details of automation tools used in the process. | Methods/Study selection |
| Data collection process | 9 | Specify the methods used to collect data from reports, including how many reviewers collected data from each report, whether they worked independently, any processes for obtaining or confirming data from study investigators, and if applicable, details of automation tools used in the process. | Methods/Data collection |
| Data items | 10a | List and define all outcomes for which data were sought. Specify whether all results that were compatible with each outcome domain in each study were sought (e.g. for all measures, time points, analyses), and if not, the methods used to decide which results to collect. | Methods/Data collection |
|  | 10b | List and define all other variables for which data were sought (e.g. participant and intervention characteristics, funding sources). Describe any assumptions made about any missing or unclear information. | Methods/Data collection |
| Study risk of bias assessment | 11 | Specify the methods used to assess risk of bias in the included studies, including details of the tool(s) used, how many reviewers assessed each study and whether they worked independently, and if applicable, details of automation tools used in the process. | Methods/Study quality |
| Effect measures | 12 | Specify for each outcome the effect measure(s) (e.g. risk ratio, mean difference) used in the synthesis or presentation of results. | Methods/Statistical analysis |
| Synthesis methods | 13a | Describe the processes used to decide which studies were eligible for each synthesis (e.g. tabulating the study intervention characteristics and comparing against the planned groups for each synthesis (item #5)). | Methods |
|  | 13b | Describe any methods required to prepare the data for presentation or synthesis, such as handling of missing summary statistics, or data conversions. | Methods |
|  | 13c | Describe any methods used to tabulate or visually display results of individual studies and syntheses. | Methods |
|  | 13d | Describe any methods used to synthesize results and provide a rationale for the choice(s). If meta-analysis was performed, describe the model(s), method(s) to identify the presence and extent of statistical heterogeneity, and software package(s) used. | Methods/statistical analysis |
|  | 13e | Describe any methods used to explore possible causes of heterogeneity among study results (e.g. subgroup analysis, meta-regression). | Methods/statistical analysis |
|  | 13f | Describe any sensitivity analyses conducted to assess robustness of the synthesized results. | Not applicable |
| Reporting bias assessment | 14 | Describe any methods used to assess risk of bias due to missing results in a synthesis (arising from reporting biases). | Not applicable |
| Certainty assessment | 15 | Describe any methods used to assess certainty (or confidence) in the body of evidence for an outcome. | Not applicable |
| **RESULTS** | | |  |
| Study selection | 16a | Describe the results of the search and selection process, from the number of records identified in the search to the number of studies included in the review, ideally using a flow diagram. | Results/1^st^ paragraph |
|  | 16b | Cite studies that might appear to meet the inclusion criteria, but which were excluded, and explain why they were excluded. | Results/1^st^ paragraph |
| Study characteristics | 17 | Cite each included study and present its characteristics. | Supplementary table 2 |
| Risk of bias in studies | 18 | Present assessments of risk of bias for each included study. | Supplementary table 2 and 4 |
| Results of individual studies | 19 | For all outcomes, present, for each study: (a) summary statistics for each group (where appropriate) and (b) an effect estimate and its precision (e.g. confidence/credible interval), ideally using structured tables or plots. | Results |
| Results of syntheses | 20a | For each synthesis, briefly summarise the characteristics and risk of bias among contributing studies. | Results and supplementary table 4 |
|  | 20b | Present results of all statistical syntheses conducted. If meta-analysis was done, present for each the summary estimate and its precision (e.g. confidence/credible interval) and measures of statistical heterogeneity. If comparing groups, describe the direction of the effect. | Results |
|  | 20c | Present results of all investigations of possible causes of heterogeneity among study results. | Not applicable |
|  | 20d | Present results of all sensitivity analyses conducted to assess the robustness of the synthesized results. | Not applicable |
| Reporting biases | 21 | Present assessments of risk of bias due to missing results (arising from reporting biases) for each synthesis assessed. | Not applicable |
| Certainty of evidence | 22 | Present assessments of certainty (or confidence) in the body of evidence for each outcome assessed. | Not applicable |
| **DISCUSSION** | | |  |
| Discussion | 23a | Provide a general interpretation of the results in the context of other evidence. | Discussion/1^st^ paragraph |
|  | 23b | Discuss any limitations of the evidence included in the review. | Discussion/limitations and strength of the study |
|  | 23c | Discuss any limitations of the review processes used. | Discussion/limitations and strength of the study |
|  | 23d | Discuss implications of the results for practice, policy, and future research. | Discussion/Implication for clinical practice and research |
| **OTHER INFORMATION** | | |  |
| Registration and protocol | 24a | Provide registration information for the review, including register name and registration number, or state that the review was not registered. | Methods/1^st^ paragraph |
|  | 24b | Indicate where the review protocol can be accessed, or state that a protocol was not prepared. | Methods/1^st^ paragraph |
|  | 24c | Describe and explain any amendments to information provided at registration or in the protocol. | Not applicable |
| Support | 25 | Describe sources of financial or non-financial support for the review, and the role of the funders or sponsors in the review. | Declarations/Funding sources and sponsors |
| Competing interests | 26 | Declare any competing interests of review authors. | Declarations/competing interests |
| Availability of data, code and other materials | 27 | Report which of the following are publicly available and where they can be found: template data collection forms; data extracted from included studies; data used for all analyses; analytic code; any other materials used in the review. | Template collection form, extracted data, data used, analytic code are available on request |

**Additional Document 2.** Literature search strategies.

Initial search on 30.06.2020

| Embase.com (1971-) | 2505 | 2485 |
| --- | --- | --- |
| Medline ALL Ovid (1946-) | 1712 | 412 |
| Web of Science Core Collection (1975-) | 1392 | 291 |
| Cochrane CENTRAL register of Trials (1992-) | 6 | 0 |
| **Total** | **5615** | **3189** |

**Embase.com 2505**

('Pseudomonas aeruginosa'/exp/mj OR 'Pseudomonas infection'/mj OR Pseudomonas/mj OR (Pseudomonas* OR P-aeruginosa*):ti) AND ('contact examination'/exp OR 'outbreak'/de OR 'disease transmission'/de OR 'bacterial transmission'/de OR 'epidemic'/de OR 'endemic disease'/de OR 'hospital infection'/de OR 'cross infection'/de OR ((contact* NEAR/3 (detect* OR examinat* OR trac* OR search*)) OR outbreak* OR transmission* OR epidemic* OR Nosocomial* OR endemic* OR ((hospital* OR cross) NEAR/3 infection*)):ab,ti) AND ('multidrug resistance'/de OR 'drug resistance'/de OR 'antibiotic resistance'/exp OR 'antibiotic sensitivity'/de OR 'extensive drug resistance'/de OR 'carbapenem resistant Pseudomonas aeruginosa'/de OR 'multidrug resistant Pseudomonas aeruginosa'/de OR ((resistan* OR sensitiv*) NEAR/3 (multidrug* OR multi-drug* OR imipenem* OR meropenem* OR carbapenem* OR beta-lactam* OR antibiot* OR anti-biot* OR antimicrob* OR anti-microb*)):ab,ti)

**Medline ALL Ovid 1712**

(*Pseudomonas aeruginosa/ OR *Pseudomonas Infections/ OR *Pseudomonas/ OR (Pseudomonas* OR P-aeruginosa*).ti.) AND (Contact Tracing/ OR Disease Outbreaks/ OR exp Disease Transmission, Infectious/ OR Epidemics/ OR Endemic Diseases/ OR Cross Infection/ OR ((contact* ADJ3 (detect* OR examinat* OR trac* OR search*)) OR outbreak* OR transmission* OR epidemic* OR Nosocomial* OR endemic* OR ((hospital* OR cross) ADJ3 infection*)).ab,ti.) AND (Drug Drug Resistance/ OR Drug Resistance, Microbial/ OR Drug Resistance, Multiple, Bacterial/ OR ((resistan* OR sensitiv*) ADJ3 (multidrug* OR multi-drug* OR imipenem* OR meropenem* OR carbapenem* OR beta-lactam* OR antibiot* OR anti-biot* OR antimicrob* OR anti-microb*)).ab,ti.)

**Web of Science Core Collection 1392**

TI=((Pseudomonas* OR P-aeruginosa*)) AND TS=((((contact* NEAR/2 (detect* OR examinat* OR trac* OR search*)) OR outbreak* OR transmission* OR epidemic* OR Nosocomial* OR endemic* OR ((hospital* OR cross) NEAR/2 infection*))) AND (((resistan* OR sensitiv*) NEAR/2 (multidrug* OR multi-drug* OR imipenem* OR meropenem* OR carbapenem* OR beta-lactam* OR antibiot* OR anti-biot* OR antimicrob* OR anti-microb*))))

**Cochrane CENTRAL register of Trials 6**

((Pseudomonas* OR P-aeruginosa*):ti) AND (((contact* NEAR/3 (detect* OR examinat* OR trac* OR search*)) OR outbreak* OR transmission* OR epidemic* OR Nosocomial* OR endemic* OR ((hospital* OR cross) NEAR/3 infection*)):ab,ti) AND (((resistan* OR sensitiv*) NEAR/3 (multidrug* OR multi-drug* OR imipenem* OR meropenem* OR carbapenem* OR beta-lactam* OR antibiot* OR anti-biot* OR antimicrob* OR anti-microb*)):ab,ti)

**Updated search on 12.01.2022**

| Embase.com (1971-) | 2805 | 1341 |
| --- | --- | --- |
| Medline ALL Ovid (1946-) | 1906 | 1902 |
| Web of Science Core Collection (1975-) | 1661 | 339 |
| Cochrane CENTRAL register of Trials (1992-) | 9 | 1 |
| **Total** | **6381** | **3583** |

*New references: 410*

**Embase.com**

('Pseudomonas aeruginosa'/exp/mj OR 'Pseudomonas infection'/mj OR Pseudomonas/mj OR (Pseudomonas* OR P-aeruginosa*):ti) AND ('contact examination'/exp OR 'outbreak'/de OR 'disease transmission'/de OR 'bacterial transmission'/de OR 'epidemic'/de OR 'endemic disease'/de OR 'hospital infection'/de OR 'cross infection'/de OR ((contact* NEAR/3 (detect* OR examinat* OR trac* OR search*)) OR outbreak* OR transmission* OR epidemic* OR Nosocomial* OR endemic* OR ((hospital* OR cross) NEAR/3 infection*)):ab,ti) AND ('multidrug resistance'/de OR 'drug resistance'/de OR 'antibiotic resistance'/exp OR 'antibiotic sensitivity'/de OR 'extensive drug resistance'/de OR 'carbapenem resistant Pseudomonas aeruginosa'/de OR 'multidrug resistant Pseudomonas aeruginosa'/de OR ((resistan* OR sensitiv*) NEAR/3 (multidrug* OR multi-drug* OR imipenem* OR meropenem* OR carbapenem* OR beta-lactam* OR antibiot* OR anti-biot* OR antimicrob* OR anti-microb*)):ab,ti)

**Medline ALL Ovid**

(*Pseudomonas aeruginosa/ OR *Pseudomonas Infections/ OR *Pseudomonas/ OR (Pseudomonas* OR P-aeruginosa*).ti.) AND (Contact Tracing/ OR Disease Outbreaks/ OR exp Disease Transmission, Infectious/ OR Epidemics/ OR Endemic Diseases/ OR Cross Infection/ OR ((contact* ADJ3 (detect* OR examinat* OR trac* OR search*)) OR outbreak* OR transmission* OR epidemic* OR Nosocomial* OR endemic* OR ((hospital* OR cross) ADJ3 infection*)).ab,ti.) AND (Drug Drug Resistance/ OR Drug Resistance, Microbial/ OR Drug Resistance, Multiple, Bacterial/ OR ((resistan* OR sensitiv*) ADJ3 (multidrug* OR multi-drug* OR imipenem* OR meropenem* OR carbapenem* OR beta-lactam* OR antibiot* OR anti-biot* OR antimicrob* OR anti-microb*)).ab,ti.)

**Web of Science Core Collection**

TI=((Pseudomonas* OR P-aeruginosa*)) AND TS=((((contact* NEAR/2 (detect* OR examinat* OR trac* OR search*)) OR outbreak* OR transmission* OR epidemic* OR Nosocomial* OR endemic* OR ((hospital* OR cross) NEAR/2 infection*))) AND (((resistan* OR sensitiv*) NEAR/2 (multidrug* OR multi-drug* OR imipenem* OR meropenem* OR carbapenem* OR beta-lactam* OR antibiot* OR anti-biot* OR antimicrob* OR anti-microb*))))

**Cochrane CENTRAL register of Trials**

((Pseudomonas* OR P-aeruginosa*):ti) AND (((contact* NEAR/3 (detect* OR examinat* OR trac* OR search*)) OR outbreak* OR transmission* OR epidemic* OR Nosocomial* OR endemic* OR ((hospital* OR cross) NEAR/3 infection*)):ab,ti) AND (((resistan* OR sensitiv*) NEAR/3 (multidrug* OR multi-drug* OR imipenem* OR meropenem* OR carbapenem* OR beta-lactam* OR antibiot* OR anti-biot* OR antimicrob* OR anti-microb*)):ab,ti)

**Additional Figure 1.** Year of publication of the included studies (N=126)


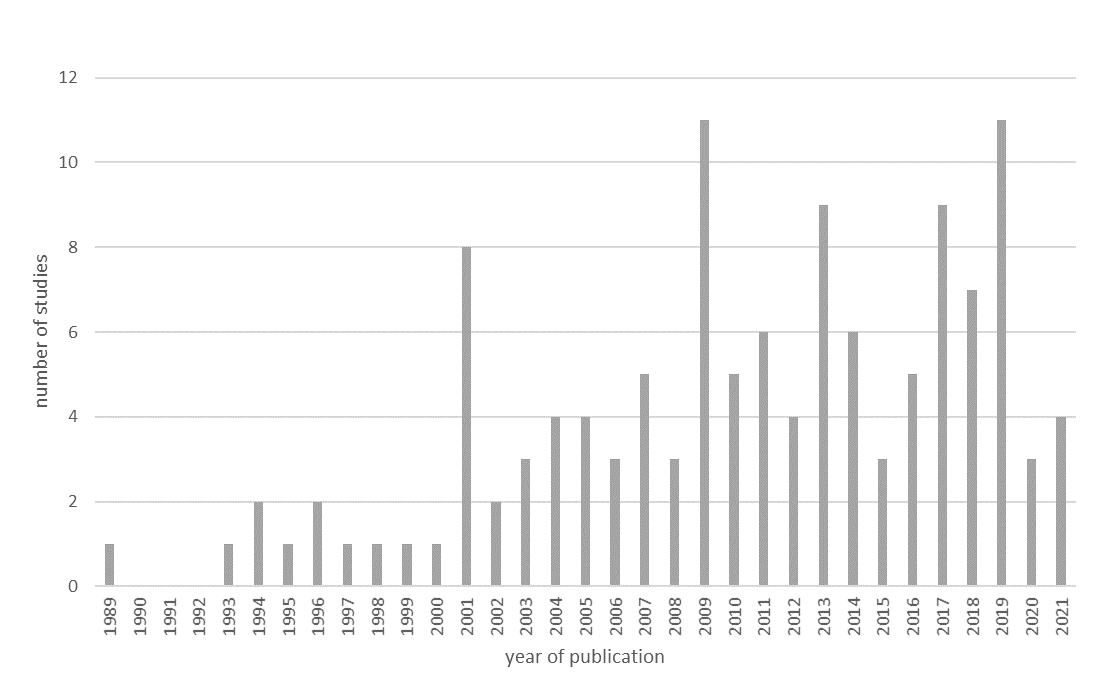

Supplement: Supplementary file 1 — Additional file 1. Table 1. Overview table of the evidence and recommendation for multidrug-resistant Pseudomonas aeruginosa in the healthcare setting, adapted from the ESCMID guidelines for the management of the infection control measures to reduce transmission of multidrug-resistant Gram-negative bacteria in hospitalized patients. Table 2. Study characteristics of the included 126 studies. Table 3. Outbreak investigations after detection of CRPA stratified by hospital setting. Table 4. Quality assessments of the included studies by study type. Document 1. PRISMA 2020 Checklist. Document 2. Literature search strategies. Figure 1. Year of publication of the included studies (N=126). [file 13756_2023_1223_MOESM1_ESM.docx]
